# Soft-Matter Confinement Modulates Excited-State Dynamics of Ru Photocatalysts for Hydrogen Evolution in Aqueous Media

**DOI:** 10.1021/acsmaterialsau.5c00212

**Published:** 2026-02-23

**Authors:** Jens H. Tran, Nikita Vashistha, Akuila Edwards, Alexander K. Mengele, Alina Koba, Sana Ullah, Jens Bauer, Jan Griebel, Michael Schmitt, Samir F. El-Mashtoly, Jürgen Popp, Dirk Ziegenbalg, Benjamin Dietzek-Ivanšić, Sven Rau, Montaha Anjass

**Affiliations:** † Institute of Inorganic Chemistry I, 9189 Ulm University, Albert-Einstein-Allee 11, Ulm 89081, Germany; ‡ 9378 Leibniz Institute of Photonic Technology, e. V. Jena, Albert-Einstein-Straße 9, Jena 07745, Germany; § Institute of Physical Chemistry, Friedrich-Schiller-University Jena, Helmholzweg 4, Jena 07743, Germany; ∥ Institute of Chemical Engineering, Ulm University, Albert-Einstein-Allee 11, Ulm 89081, Germany; ⊥ Leibniz Institute of Surface Engineering (IOM), Permoserstraße 15, Leipzig 04318, Germany; # Abbe Center of Photonics, Albert-Einstein-Straße 6, Jena 07745, Germany; ∇ Department of Chemistry, University of Sharjah, Sharjah 27272, United Arab Emirates

**Keywords:** electrospun nanofibers, molecular dyads, photochemical molecular devices, photocatalysis, hydrogen evolution reaction (HER), femtosecond transient absorption spectroscopy

## Abstract

The development of photochemical molecular devices (PMDs) is essential for the mechanistic understanding of solar-to-fuel conversion, yet translation from homogeneous solution to heterogeneous systems remains largely unexplored, as molecular confinement may lead to unpredictable reactivity and dynamics. Here, we report Ru-based photosensitizer—bridging ligand—Rh/Pt catalyst assemblies, as well-characterized model systems, immobilized within electrospun polyacrylonitrile (PAN) nanofibers as a soft-matter matrix for light-driven hydrogen evolution in fully aqueous media. Structural and spectroscopic analysis (SEM, XPS, EDX, ATR-IR, FT-Raman, O-PTIR, NMR, UV–vis and diffuse reflectance) confirmed homogeneous distribution and chemical integrity of the molecular components after they have been embedded into the nanofibrous soft-matter matrix. Steady-state emission revealed that the polymer matrix alters the local environment around the complex, destabilizing the π* orbital energy of the tpphz bridge. Emission and femtosecond transient absorption (fsTA) spectroscopy further demonstrated that the surface and buried complexes give different contributions to excited-state dynamics, which is significantly different from what was observed for solution. Light-driven hydrogen evolution experiments confirmed the activity of all fibers containing the catalytic Rh or Pt centers. The observable photocatalytic activity is determined by the nature of the catalytic center and is only insignificantly altered upon immobilization into the PAN matrix. The nanofibers enable stable photocatalysis with environmentally benign sacrificial donors, suppress leaching, and preserve the molecular integrity under catalytic turnover. These findings highlight how a soft-matter environment modulates the photophysical pathways of molecular photocatalysts and provides mechanistic insight into designing fiber-supported systems for sustainable hydrogen evolution in aqueous media.

## Graphical abstract

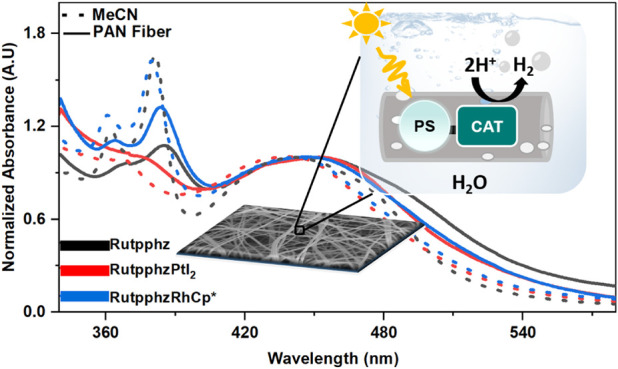

## Introduction

In the face of anthropogenic climate change, the quest for sustainable, clean energy sources has led to the exploration of renewable energy technologies. Among renewable sources, solar energy represents the most abundant and universally accessible resource,^1^ delivering nearly four magnitudes more solar energy to the earth’s surface than is consumed per year worldwide.^2,3^ While photovoltaic methods convert solar energy directly into electric energy, complementary molecular-level approaches, such as photoelectrochemical cells^4–12^ or photochemical molecular devices (PMDs),^13–16^ are capable of direct solar-to-chemical energy transformation. Recent progress in this field has considerably advanced our understanding of light-driven molecular transformations.^17–24^ To bridge the gap between molecular understanding and scalable application, we report the incorporation of well-understood Ru complexes into scalable electrospun polyacrylonitrile (PAN) soft-matter matrices.^25^ In particular, molecular dyads incorporating RhCp*Cl or MX_2_ (Cp*=pentamethylcyclopentadienyl; M = Pt,Pd; X = Cl,I) as catalytic centers, linked by bridging ligands such as tpphz (tetrapyrido­[3,2-a:2′,3′-c:3″,2″h:2′″,3′″-j]­phenazine) or 2,5-tpy (2,2’:5′,2’’-terpyrid-ine), are promising candidates to perform solar energy conversion.^18,20,22,26–28^ The report of Mengele et al. featured the dinuclear complex [(tbbpy)_2_Ru­(tpphz)­Rh­(Cp*)­Cl]-Cl­(PF_6_)_2_ (RutpphzRhCp*), maintaining catalytic hydrogen production for up to 650 h.^18^ The dinuclear complex [(tbbpy)_2_Ru­(tpphz)­PtI_2_]­(PF_6_)_2_ (RutpphzPtI_2_) possessing a similar ligand architecture but equipped with a different catalytic center was reported with a turnover number (TON) of 520 for hydrogen production. Notably, an active repair mechanism enabling full recovery of the catalytic activity was reported—a particularly remarkable feature for long-term applications based on molecular devices.^19,29^

To date, most PMDs have primarily been studied in solution and, thus, struggle with limited reusability of the catalyst and ineffective phase separation of reactants. Immobilization of the active species within a solid matter offers a promising strategy to circumvent these challenges.^4–6,30,31^ In this context, the choice of a suitable host matrix is just as important as synthesizing molecules capable of harvesting a broad range of the solar spectrum.^10–12^ With their exceptional recyclability, scalability, numerous active sites, porous structure, and high stability, photocatalytic fibers are thought to be among the most reliable and effective materials for energy conservation, wastewater treatment, and air purification.^32–35^ In recent years, electrospinning has emerged as a versatile and cost-effective technique for fabricating nanofibers, demonstrating remarkable potential in clean energy applications.^36–38^ The medium-polar polymer polyacrylonitrile (PAN) employed in this work is known to influence the electronic and optical properties of embedded molecular components.^32,39,40^ Furthermore, in 2022, Stepping et al. reported that [Ru­(bpy)_3_]^2+^ embedded into PAN fibers encounters distinct microenvironments within the fiber,^41^ highlighting both the importance and difficulty of understanding the photophysical behavior of photocatalysts in such soft-matter matrices. Lin et al. demonstrated the potential of such a design using a poly­(ethylene oxide)/polyvinylpyrolidinone polymer blend embedding donor–acceptor heterojunctions, boosting photocatalytic activity by a factor of 34 and attributing this to improved exciton dissociation.^42^

Herein, we report the synthesis of electrospun PAN nanofibers and embedding, in total, three Ru complexes: [(tbbpy)_2_Ru­(tpphz)]­(PF_6_)_2_ (Rutpphz) or its dinuclear derivatives RutpphzPtI_2_ and RutpphzRhCp*, along with the investigation of their structural, morphological, and photophysical properties. We elucidated the influence of soft-matter confinement on their photophysical and catalytic behavior by systematically probing their steady-state and time-resolved optical properties. We also demonstrate the potential of these embedded systems for photocatalytic hydrogen evolution reactions (HER) in aqueous media. These findings provide new insights into the design of functional hybrid materials and mark a step forward toward the development of efficient solid-state photocatalytic devices.

## Results and Discussion

### Fibers Preparation and Characterization

The electrospun nanofibers were prepared using a Fluidnatek, LE-50 instrument operated in a horizontal setup. A 14-gauge needle was used, and aluminum foil-covered collector was employed. Electrospinning parameters included a needle-to-collector distance of 15 cm, an applied voltage of 13 kV, and a flow rate of 500 μL h^–1^. Spinning solution (total mass of 5 g), consisting of 0.25 g of PAN and 0.031 g of the respective Ru complex dissolved in dimethylformamide (DMF), was stirred for 48 h. Electrospinning of the PAN solution containing the Ru-complexes yielded uniform, orange-colored nanofiber mats with a theoretical Ru-complexes loading of 5.9 wt %. Figure 1 shows the schematics of the electrospinning process and the SEM image of the resulting nanofiber. All three Ru complexes Rutpphz, RutpphzPtI_2_, and RutpphzRhCp* were successfully immobilized within the polymer scaffold without observable macroscopic aggregation.

**Figure 1:**
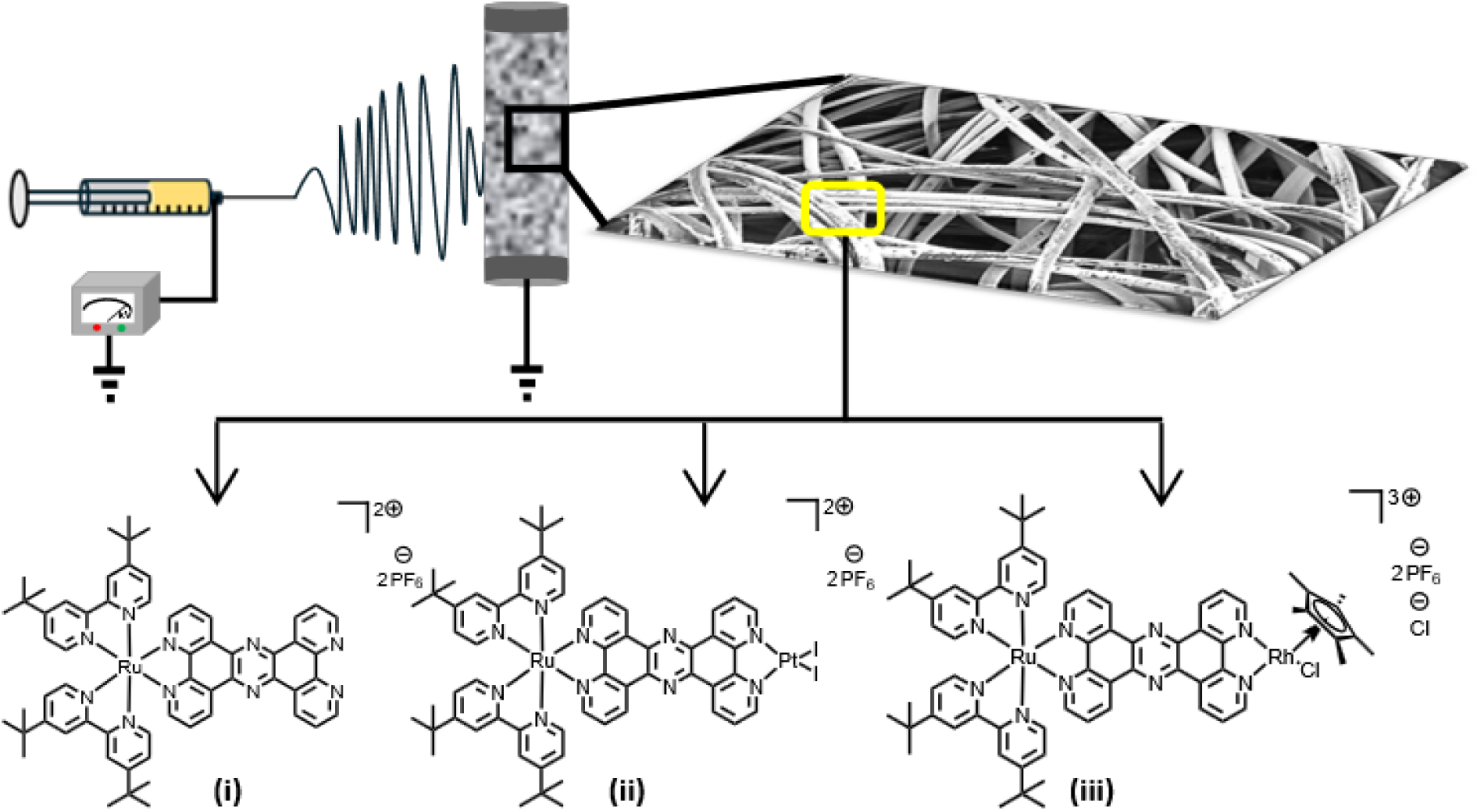
Schematic of the electrospinning process used to fabricate PAN nanofibers embedded with three different Ru-complexes: (i) Rutpphz, (ii) RutpphzPtI_2_, and (iii) RutpphzRhCp*. Each set of PAN fibers contains one of these Ru complexes. The chemical structure of the Ru complexes and the SEM image of the resulting nanofibers are also shown.

The morphology of the fibers was analyzed by scanning electron microscopy (SEM) (see ESI, Figure S1) acquired on a Thermo Fisher Apreo2 at an acceleration voltage of 5 kV (spot size: 0.4 nA). Beam deacceleration was applied to improve the image quality by using a stage bias of 600 V. Samples were mounted on aluminum stubs with carbon tape. The average fiber diameters (*N* > 20) were determined as 860 ± 640 nm for Rutpphz, 860 ± 440 for RutpphzPtI_2_, 520 ± 100 nm for RutpphzRhCp*, and 484 ± 99 nm for pure PAN. These results indicate that the incorporation of different complexes slightly influences fiber formation. The chemical composition of the fibers was verified with EDX, XPS, NMR, IR, and UV–vis (see ESI, Tables S1–3, S4–8, Figures S6, S11, S12 and S19–21).

XPS measurements are performed on Kratos Ultra DLD using monochromic Al K_α_ radiation at 1486.6 eV at room temperature with a background pressure of 1 × 10^–7^ Pa. Spectra are acquired from an ∼ 220 μm analysis area under normal incidence configuration and charge neutralization. The UNIFIT 2025 software is used for spectra analysis and composition calculation considering the specific transmission correction of the XPS machine. For curve fitting convolved Gaussian–Lorentzian peak profiles are simultaneously optimized with a Shirley background profile. The spectral calibration is set to the aromatic sp^2^ carbon peak in the C 1s core-level spectrum at 284.7 eV. EDX spectra were recorded in a Zeiss Gemini Ultra 55 SEM (Bruker XFlash 6–30 detector) at a beam voltage of 5 kV, a beam aperture of 30 μm, and a working distance of 5.3 mm. The spectra were accumulated over a bunch of fibers on an area of 38.6 μm × 29.0 μm for 14 h. Both XPS (see ESI Table S1–3) and EDX (see ESI Table S4–7) confirm the presence of Ru in all fibers and, in the case of RutpphzPtI_2_ and RutpphzRhCp*, also Pt, I, Rh, and Cl, indicating the integrity of the molecular components embedded into the PAN fibers. Also, the observed element ratios of the Ru-complexes in XPS and EDX were consistent with the theoretical values. The XPS data verify the C content in the expected ranges but show lower values for the N content and higher contribution of O, indicating hydrolysis of the polymer backbone, in accordance with the composition found in EDX. The C values found by EDX are slightly higher than found by XPS. This is attributed to the correction of the background and X-ray reabsorption performed for EDX. The Bruker ESPRIT software used provides established physical models for data evaluation based on flat surface samples and well-defined measured geometry. Crucial experimental parameters such as applied electron energy, take of angle (i.e., tilt angle between sample surface and EDX detector) are not accounted for in the nanofibers. The 3D nature, uneven distribution and arbitrary alignment of the nanofibers lead to uncertainties in these parameters and therefore result in the observed deviations of the composition. Even at the low beam voltage of 5 kV, the spatial resolution of EDX is about a micrometer, thus exceeding the nanofiber thickness. Therefore, the fiber is probed as a whole by EDX. Fibers were also probed with vibrational methods: attenuated total reflectance infrared (ATR-IR), Raman and optical photothermal infrared (O-PTIR) spectroscopy. Characteristic vibrational modes were identified with ATR-IR and Raman spectroscopy, while mapping of the fibers was performed with O-PTIR (see below). ATRIR (recorded using a diamond ATR crystal under dry-air purge, averaging 500 scans per sample) can probe the region below 980 cm^–1^ which is unaccessible by O-PTIR, showing the characteristic vibration of the PF_6_^–^ anion at 845 cm^–1^, without indication for any degradation products such as PF_3_ or PF_3_O (see ESI Figure S6).^43,44^ Raman spectroscopy provided complementary information exhibiting vibrations in the fingerprint region associated with the Ru-complexes (for details see ESI vibrational spectroscopy). Also, the NMR spectra obtained by dissolving the fibers in dmf-d^7^ show the PF_6_^–^ anion. The ^19^F-NMR spectra show a doublet at 71.7 ppm (^3^J_P–F_ = 709 Hz) characteristic for PF_6_^–^, also without indication of PF_3_O or PF_3_. ^31^P NMR were also taken, however showed no signal, probably due to the overall low concentration and lower sensitivity of ^31^P compared to ^19^F. Diffuse reflectance was employed to verify the integrity of the embedded chromophores. Diffuse reflectance spectra (taken on a UV-2600 UV–vis spectrophotometer, Shimadzu, Japan) of the fibers were measured against BaSO_4_ as a reference. Fibers were left on the aluminum foil after preparation and were clamped between two microscopy slides. The reflectance spectra resemble the absorption spectra of the native Ru complexes (see ESI Figure S11).

UV–vis spectroscopy constitutes a complementary and more sensitive tool to probe the chromophoric Ru-complexes within the fibers. For this analysis, the fibers were dissolved in DMF, and the amount of Ru-complex was determined from the visible absorption maximum. Assuming that the total mass is the sum of PAN and the respective Ru-complex, the loading of all embedded complexes was determined as 6.5–6.6 wt %, in good agreement with the theoretical value of 5.9 wt % (details in ESI Table S8). Notably, the absorption spectra of the dissolved fibers were identical to those of the Ru-complexes in DMF solution (see ESI Figure S7), in accordance with diffuse reflectance, indicating that the chromophores remained chemically intact during electrospinning. The dielectric constant of the two environments is comparable (ε_DMF_ = 36.7 and, for an ideal system, ε_DMF+PAN_ = 40) and is a weighted sum of ε_DMF_ and ε_PAN_. In contrast to XPS or EDX, UV–vis quantification is fast, simple, and benefits from the high molar absorption coefficients of the chromophores, allowing precise and reproducible determination of Ru complex incorporation into the nanofibers.

Complementary to the elemental analysis provided by XPS and EDX, vibrational spectroscopy was employed to get a molecular-level insight into the chemical composition and structure of materials. For this purpose, FT-Raman spectroscopy (Figure S6a) was first applied to obtain bulk spectra of the electrospun PAN fibers and to identify Raman bands distinct from those of the PAN matrix, potentially indicative of the embedded Ru complexes. FT-Raman uses near-infrared excitation at 1064 nm and is therefore largely free of emissive interference, making it particularly suitable for materials that exhibit strong background signals under visible excitation. However, FT-Raman does not allow for spatially resolved imaging. To achieve chemical imaging, Raman mapping at 785 nm was attempted, but this approach was severely hampered by intense phosphorescence from the sample. Consequently, we turned to O-PTIR spectroscopy, which combines infrared absorption contrast with submicron spatial resolution. O-PTIR successfully confirmed and spatially resolved the distribution of the Ru-complexes within the PAN nanofibers, effectively overcoming the fluorescence limitations of visible Raman microscopy. The molecular specificity and submicron spatial resolution of O-PTIR enabled detailed assessment of the fibers’ compositional uniformity and structural homogeneity. A pointwise mapping approach was employed, in which each spectrum in Figure 2 corresponds to a single spatial measurement across the fiber. The color of each spectrum matches the color scale on the O-PTIR image, where orange indicates the point of highest signal intensity and blue corresponds to the lowest intensity region. The spectra of pristine PAN nanofibers were consistent with the literature, exhibiting characteristic bands at 2937 and 2869 cm^–1^ (C–H stretching), a dominant peak at 2240 cm^–1^ (C≡N stretching), 1667 cm^–1^ (C=O/C=N stretching)^45,46^ 1453 cm^–1^ (CH_2_ bending), 1360 cm^–1^ (CH_2_ wagging), and 1073 cm^–1^ (C–C backbone stretching.^45^

**Figure 2:**
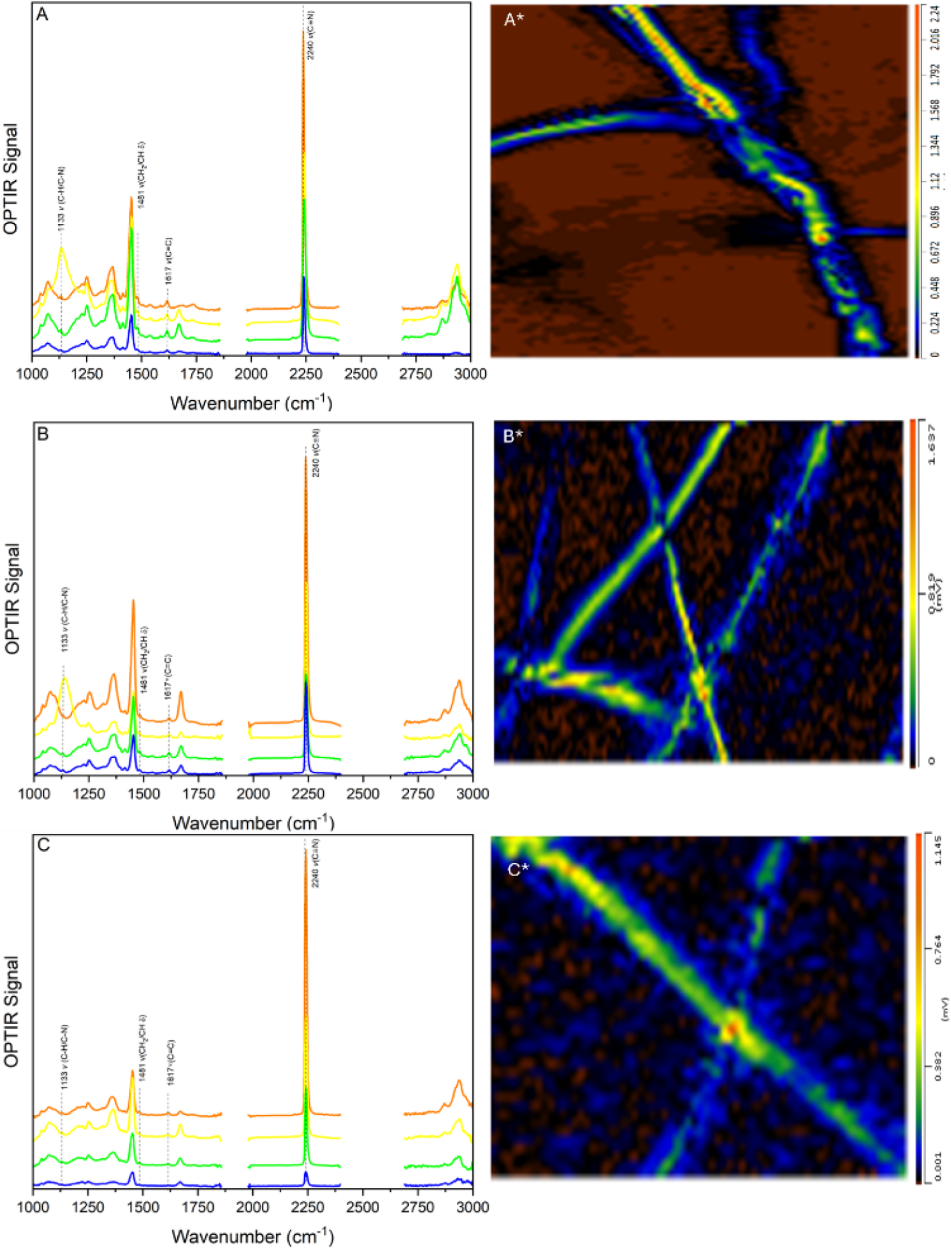
O-PTIR intensity maps of electrospun PAN fibers containing (A) Rutpphz, (B) RutpphzPtI_2_, and (C) RutpphzRhCp*, recorded at 2245 cm^–1^ (C≡N stretch of PAN). Corresponding pointwise spectra and images are indicated with *. Each spectrum represents a single-point measurement, with color indicating relative signal intensity (orange = high, blue = low). Representative spectra from selected points are shown next to each map. Measurements were performed using 40×, 0.78 NA Cassegrain objective. The characteristic aromatic C=C/C=N stretching band at 1617 cm^–1^ confirms the presence of the Ru-complexes, demonstrating their successful incorporation within the PAN nanofibers.

Spectral comparison between pure PAN fibers and those containing the Ru-complexes revealed additional ligand-specific bands associated with the tbbpy ligand, including features at 1133 and 1415 cm^–1^ (C–H bending/C–N stretching), 1481 cm^–1^ (CH_2_/C–H bending), and 1617 cm^–1^ (aromatic C=C stretching). A minor band at 1413 cm^–1^, absent in the mononuclear complex, likely arises from metal–ligand coordination effects. The bands at 1133, 1481, and 1617 cm^–1^ correspond well with the resonance Raman data reported by Tschierlei et al.^47^ for the tbbpy ligand. Although the 1617 cm^–1^ vibration has been previously attributed exclusively to [Ru­(tbbpy)_3_]^2+^, its presence across all complexes studied here suggests it may have a strongly IR-active mode of an aromatic ring along the ligand backbone. The persistence of this 1617 cm^–1^ band in all Ru complexes fibers, free from overlap with PAN vibrations, serves as a robust marker for the incorporation of the Ru-complexes.

The spectral consistency across multiple measurement points as seen in Figure 2 supports homogeneous incorporation of the Ru-complexes within the PAN nanofibers. Such uniformity is crucial for efficient charge transport and maximizing catalytic site accessibility—both key factors for enhanced HER performance.

In summary, the combination of EDX, NMR, UV–vis, XPS, FT-IR, ATR-IR, and O-PTIR gives a consistent and reliable picture of the fiber composition; the Ru complexes were successfully incorporated with the expected loading, retained their chromophoric integrity, and maintained their structural identity during electrospinning.

To assess how embedding the molecular components in PAN fibers affects the optical properties and to verify chromophore integrity before/after catalysis, we recorded steady-state absorption and emission spectra of the fiber mats. Absorption spectra were measured on a JASCO V-780, and emission spectra were measured on an Edinburgh Instruments FLS980, collected in reflection mode from samples in a quartz cell (10 mm path length). UV–vis spectra acquired in a glovebox were recorded with an Avantes AvaSpec-ULS2048CL detector and an AvaLight-DH-S-BAL light source via fiber-optic coupling. Figure 3a shows normalized steady-state UV–vis absorption spectra of the Ru-complexes in MeCN solution and embedded within the electrospun nanofiber. The absorption behavior features a characteristic metal-to-ligand charge transfer (^1^MLCT) band corresponding to Ru­(dπ) to (tbbpy/tpphz)­π* transitions located at 451, 452, and 446 nm for Rutpphz, RutpphzPtI_2_, and RutpphzRhCp*-based fibers, respectively.^48,49^ The sharp bands between 360 and 420 nm are attributed to n→ π* or π→ π* transitions of the tpphz bridging ligand.^50^ The absorption behavior of these Ru-complexes, homogeneously dissolved in MeCN, shows no difference in the MLCT absorption maxima (442 nm), consistent with observations reported in a previous study.^18^ Upon immobilization in the nanofibers, a red shift of the absorption maxima of Rutpphz, RutpphzPtI_2_ and RutpphzRhCp* by 0.05 eV, 0.06 eV, and 0.02 eV, respectively, is observed. This is likely due to aggregation of the complex within the nanofiber. In addition, all nanofibers exhibit spectral broadening of the MLCT band, attributed to increasing intermolecular interactions. Notably, this spectral broadening persists even after the Ru-complex-loaded fibers are dissolved in DMF (see ESI Figure S7), suggesting that the polymer matrix alters the local environment around the embedded complexes. Other effects may also contribute, including aggregation during fiber formation, residual polymer–complex interactions, or heterogeneous solvation environments.

**Figure 3:**
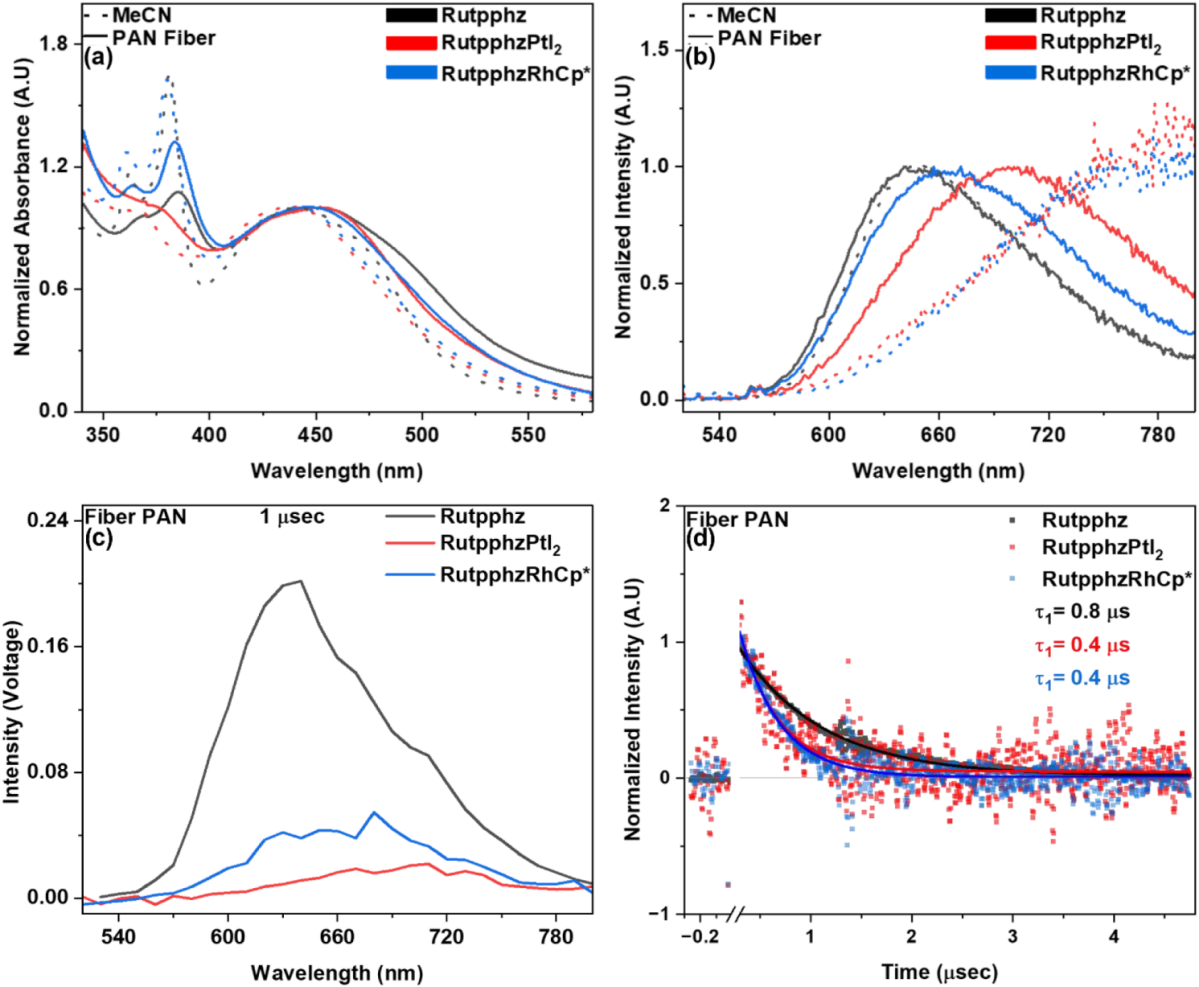
Normalized (a) steady-state UV–vis absorption and (b) emission of Rutpphz, RutpphzPtI_2_ and RutpphzRhCp* in MeCN and PAN nanofiber, (c) comparative emission spectra of the Ru-complexes embedded into nanofiber at 1 μs delay time, and (d) normalized emission decay profile of Rutpphz, RutpphzPtI_2_ and RutpphzRhCp* in PAN nanofiber.

Figure 3b represents the normalized emission from the Ru complexes in MeCN solution and nanofiber. As reported earlier, in complexes with tpphz bridging ligand, the emissive MLCT state is located at the phenanthroline part of the bridging ligand.^50^ The addition of a heavy metal catalytic center (such as Pt or Rh) lowers the π* energies of tpphz.^18^ Due to this, we observe a bathochromic shift in the emission maxima of the fibers from 648 nm for Rutpphz to 785 nm for both RutpphzPtI_2_ and RutpphzRhCp* in MeCN solution. When the complexes are embedded within the nanofibers, a blue shift in emission maxima is observed by 0.02 eV, 0.20 eV, and 0.29 eV for Rutpphz, RutpphzPtI_2_, and RutpphzRhCp*, respectively, indicating that the π* energy levels of the tpphz ligand are less stabilized in the fiber environment compared to solution (MeCN). Limited structural relaxation of the excited states inside the fibrous material might be responsible for this effect. Dissolving the fibers in DMF (see ESI, Figure S7) reveals that only RutpphzPtI_2_ retains a blue shift in emission, suggesting strong polymer–complex interactions that destabilize and raise the energy of the π* orbitals of the tpphz ligand. Figure 3c compares emission from the nanofiber samples at 1 μs delay time. The decrease in emission intensity might be due to the formation of nonluminescent charge-separated (CS) states resulting from the transfer of the electron from the excited Ru center to the Rh or Pt center, respectively.^51,52^ However, in a nanofiber, the high local density of Ru-complexes will also affect the emission intensity. Our calculations (see ESI, Table S4) show that the concentrations of RutpphzPtI_2_ and RutpphzRhCp* are 77% and 54% lower in the fibers, with respect to Rutpphz, leading to correspondingly fewer emitting molecules. Thus, a reduction in emission intensity proportional to the reduction in the number of emitting molecules is expected. However, RutpphzPtI_2_ exhibits a 90% decrease in emission intensity, while RutpphzRhCp* shows 79% decrease. This additional decay can be attributed to electron transfer across the tpphz bridge or may be the emission originating solely from complexes buried inside the fiber, whose orientation hinders electron transfer. Figure 3 (d) compares the normalized emission decay kinetics of the three complexes. The decay profile is fitted by a monoexponential fit. Upon addition of a heavy metal catalytic center, the radiative decay rate of carrier density at phenanthroline is 2 times faster than in the Rutpphz complex, indicating photoinduced electron transfer in the presence of a catalytic center. The rate of photoinduced electron transfer is 1.25 × 10^6^ s^–1^ for the nanofibers containing Ru complexes with either Pt or RhCp* as the catalytic center. In comparison, the previously reported rate for RutpphzPtI_2_ is 3.05 × 10^7^ s^–1^ in aerated MeCN,^53,54^ being approximately 24 times faster. These observations suggest that the polymer fiber environment can restrict molecular motion and thus add additional barriers for electron transfer.

To investigate the photoinduced process in the embedded Ru-complexes, femtosecond transient absorption (fsTA) (see Figure 3) and nanosecond transient absorption (nsTA) (see ESI Figure S22) experiments were conducted. The transient spectra of all three complexes display ground-state bleach (GSB) and excited-state absorption (ESA) similar to those seen in the previously studied RuMX_2_ (M = Pt, Pd; X = I, Cl) and RuRhCp* complexes.^20,48,53^ The GSB forms upon the population of the metal-to-ligand charge transfer (MLCT) state, while the excited-state absorption (ESA) arises from transitions within ligand-centered excited states and/or LMCT (from reduced ligand to oxidized Ru center). Notably, the GSB maxima of the Ru-complexes do not coincide with the steady-state absorption maximum (see Figure 4a–c, inverted gray region), indicating there are relaxation processes that occur faster than the experimental observation time scale within the polymeric nanofiber matrix.

**Figure 4:**
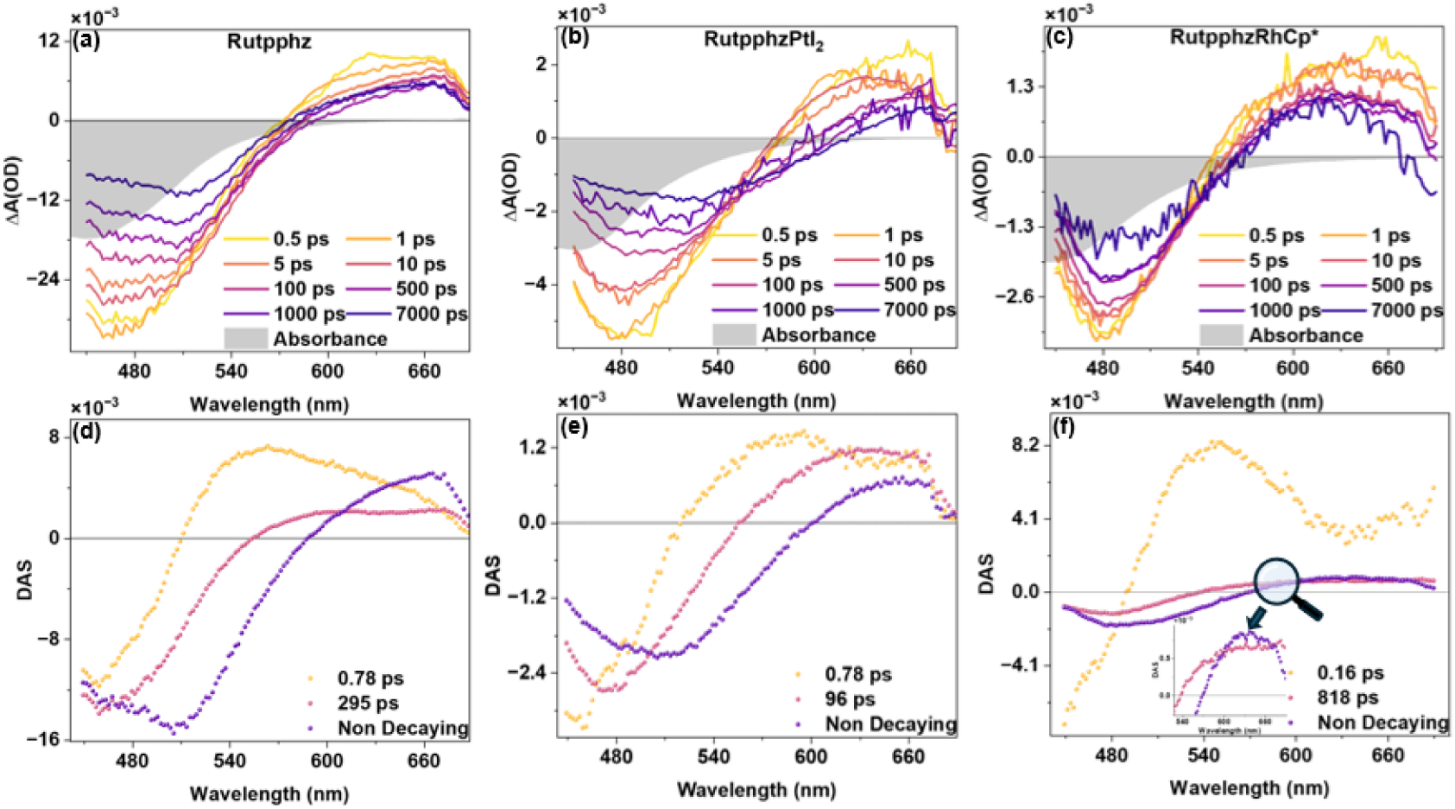
fsTA (a), (b), (c) time-dependent spectra and (d), (e), (f) decay-associated spectra (DAS) of Rutpphz, RutpphzPtI_2_ and RutpphzRhCp* in PAN nanofibers. The samples were excited with 400 nm pump, and the data were collected with a white light probe. For reference, the steady-state absorption of the complex (inverted gray area) is plotted with the spectra.

The decay of the excited state in the studied Ru-complexes is a multistep process, beginning with an ^1^MLCT transition, followed by intersystem crossing, vibrational relaxation, and the formation of a phenanthroline-centered ^3^MLCT state. From this point, the system can relax via two main pathways: an intraligand charge transfer (ILCT) to the phenazine unit, which can result in rapid nonradiative decay to the ground state or promote electron transfer to the catalytic center, resulting in a CS state. Alternatively, a radiative decay can occur directly from the phenanthroline-centered MLCT state. The relative contribution of each pathway is influenced by the polarity and rigidity of the surrounding environment. Previous literature on RuPt/RuPd dyads reported that the ILCT process transferring charge from the phenanthroline sphere to the central phenazine sphere of the tpphz bridging ligand occurs within 5 ps, while for the RhCp* dyad, this process takes 11 ps.^20,47^

Figure 4d–f represents the decay-associated spectra (DAS) of the Ru-complexes in the fibers. Contrary to solutions (where the process happens on a 1–12 ps time scale), the charge transfer to phenanthroline-centered states in the nanofibers happens in the femtosecond regime,^14,20^ much faster than can be resolved spectrally. This phenanthroline-centered state relaxes via ILCT to a phenazine-centered MLCT state in a subpicosecond time, represented by the first component in the DAS (Figure 4d–f). The transfer of charge density to phenazine is indicated by a broad excited-state absorption (ESA), centered at 560 nm (for Rutpphz and RutpphzRhCp*) and 580 nm (for RutpphzPtI_2_), corresponding to the phenazine radical anion.^53^ This radical anion buildup occurs with a significant blue shift of the ESA maxima in less than 1 ps time (Figure 4a–c), as opposed to complex in solution.^47,48^ Furthermore, in fibers, the Rutpphz complex forms the phenazine-centered state with a much shorter lifetime of only 0.78 ps, compared to previous studies in MeCN where the same state exhibited a population lifetime of 240 ps.^47^ This suggests that by simply immobilizing the complex in the fiber, this ILCT occurs at a faster rate, regardless of the Pt/Rh metal coordination. The second and third components in DAS (see Figure 4d) for the mononuclear complex will correspond to the decay of the phenazine-centered dark state. Given the significant shift in the isosbestic point and the change in spectral shape observed in the DAS, it can be inferred that the population density is delocalized across the entire tpphz ligand rather than being localized on the phenazine part of the ligand, resulting in two decay times: 295 ps and a nondecaying component.

For the dyads, the second component in the DAS (see Figure 4e,f) shows the formation of the CS state by electron transfer from the reduced phenazine species toward the second metal center, indicated by a shift of the ESA to 600 nm, decaying with an infinite lifetime (third component of DAS). However, a subensemble of molecules immobilized deep within the fiber, and thus highly restricted in reorganizing their molecular geometries, may not undergo electron transfer, leading to radiative decay of their excited-state population, as indicated by the residual phosphorescence seen in Figure 3c. For those that do support electron transfer, the ESA feature (represented by the second component of DAS) in RutpphzRhCp* corresponds to ^3^IL absorption by [(tbbpy)_2_Ru^III^tpphz^.–^Rh^III^Cp*Cl] while for RutpphzPtI_2_ this is a ^3^LMCT state.^20,47,53^ In RutpphzPtI_2_ the depopulation of phenazine-centered MLCT_Ru_ and transfer to diiodoplatinum sphere happens relatively rapidly, with a time constant of 96 ps and a rate constant of k_LMCT_ = 1.04 × 10^10^ s^–1^, whereas it is slowest in RutpphzRhCp*, occurring over 818 ps with k_LMCT_ = 1.22 × 10^9^ s^–1^.

In solution, RutpphzPtI_2_ and RutpphzRhCp* exhibit lifetimes of 340 and 450 ps, respectively, for this process.^53,54^ These observations suggest that the transition proceeds slower in the RutpphzRhCp* fibers, likely due to structural constraints imposed by the polymer, which likely increases the barrier for electron transfer to the Rh center, which in solution undergoes large structural changes upon reduction. Furthermore, the CT rate observed in fsTA is much faster than that inferred from emission measurements. This difference likely stems from the fact that emission originates mainly from buried molecules, while fsTA probes a combination of charge-transfer processes in molecules on the surface and buried within the fiber. The molecules on the surface give the fast electron transfer across the tpphz bridge. From Figure 4c, we observe that there is no significant absorption above 660 nm (especially for longer time), so we can rule out Cl dissociation upon RutpphzRhCp* irradiation as it is mentioned in a previous study that this absorption is due to (bright) Rh^II^ → tpphz (^3^MLCT) in [(tbbpy)_2_Ru^III^(tpphz)­Rh^II^Cp*].^53^

To observe the decay of this long-lived CS state, we used nsTA (see ESI Figure S22). From Figure S22a,b, we observed the decay of charge density delocalized on tpphz with a lifetime of 0.8 μs, matching the phosphorescence lifetime observed in Figure 3d. This suggests that, in the absence of the catalytic center, the final relaxation occurs through radiative decay from phenanthroline-centered state. For the samples with the catalytic center, we see no GSB signature, indicating that the final state is CS state formed after charge transfer to the catalytic center, and it completely decays in a time faster than the measurement capabilities of nsTA.

### Leaching Behavior and Verification of the Composition

Prior to evaluating the nanofibers for HER performance, we conducted leaching studies to assess the stability of the embedded Ru-complexes. This was done by immersing the electrospun nanofibers in solvents of varying polarity (toluene, THF, MeCN, MeOH, water, and aqueous sodium ascorbate) for 24 h. The solutions were then analyzed with UV–vis spectroscopy (see ESI, Figure S8–10). Among the tested solvents, significant leaching was observed mainly in MeCN and less in MeOH, consistent with the known solubility of the complexes in these solvents. An additional effect on the leaching is the swelling of PAN in MeCN.^55^ Quantification of the released fraction relative to the total complex content in the nanofiber revealed leaching of 52% for Rutpphz, 10% for RutpphzPtI_2_, and 38% for RutpphzRhCp*. Minor leaching occurred for Rutpphz and RutpphzRhCp* in MeOH, whereas no measurable leaching was observed in THF, toluene, and the aqueous solutions. Importantly, the absence of leaching in the catalytic solution, aqueous sodium ascorbate, demonstrates the HER described below to proceed heterogenously excluding contributions from leached Ru-complex into the homogeneous solution. The extent of leaching in MeCN provides an estimate for the fraction of Ru-complexes accessible at the nanofiber surface. This assumption is in accord with our previous reports showing that only surface-exposed complexes are in contact with the solvent and contribute to photocatalytic turnover.^41^ Accordingly, the measured leached fractions were used in the following to correct the TONs, assuming that only complex on the surface has contact to the solvent and therefore can leach or participate in the HER. This rationale is consistent with earlier findings on inorganic nanofiber systems, where increasing surface area correlated with enhanced activity, as exemplified by superior performance of hollow versus solid TiO_2_ nanofibers in light-induced methylene blue degradation.^56^

### Hydrogen Evolution Reaction (HER) Experiments

HER was performed using three different conditions: (i) in glass vials, heterogeneously suspending the nanofibers with embedded Ru-complexes; (ii) in glass vials, homogeneously dissolving the Ru-complexes; (iii) in a flow reactor, heterogeneously using the nanofibers with embedded Ru-complexes. (i) and (ii) were performed to compare the effect of heterogenization on the TON. (iii) serves as a proof-of-concept implementation of a reactor design for future upscaling attempts.

Heterogeneous hydrogen evolution in vials was performed using a previously reported photoreactor at 450 nm illumination and a light power of 80 mW/cm^2^ (determined according to^57^), for 24 h. The samples were prepared inside a glovebox in 4 mL borosilicate vials containing 2–3 mg of the electrospun nanofiber mats immersed in 2 mL of aqueous solution of either sodium ascorbate or ascorbic acid (100 mM). Using the same fibers with sodium ascorbate as SD in total three catalysis cycles were run. For this purpose, the fibers were washed with water (2 times, 20 mL/2 mg­(fiber)), then soaked in 20 mL water overnight and dried *in vacuo*, in between catalysis cycles. All measurements were performed as duplicates. Control measurements were performed with (i) blank nanofibers consisting of pure PAN, with similar thickness and morphology and (ii) dark measurements with exactly the same preparation but without irradiation. Neither control measurement resulted in detectable amounts of H_2_, confirming that hydrogen formation originates exclusively from light-driven processes involving the embedded complexes. The nanofibers before and after applying catalytic conditions were analyzed by SEM, UV–vis, diffuse reflectance, XPS and EDX to assess the stability of the molecular components. Illumination of all three Ru-complex-containing nanofibers, with sodium ascorbate or ascorbic acid as sacrificial donor, led to detectable amounts of H_2_. Using ascorbic acid as sacrificial donor resulted in only traces of hydrogen, below the calibration limit of the GC; therefore, these values are not further analyzed. In contrast, sodium ascorbate yielded detectable amounts of hydrogen with corrected TONs of 3 for RutpphzPtI_2_ and 1 for RutpphzRhCp*. Rutpphz nanofibers also produced H_2_, but at levels below the calibration limit of the instrument (see ESI Table S9). The significantly lower hydrogen evolution observed with ascorbic acid compared to sodium ascorbate likely stems from the pH-dependent redox behavior of the sacrificial donor. In aqueous solution, ascorbic acid predominates and is a weaker electron donor than the deprotonated ascorbate anion, thus exhibiting less favorable redox properties for the Ru-complexes. A detailed study by Natali et al.^58^ illustrated that in homogeneous photocatalytic systems, hydrogen production first plateaus at acidic pH (∼3.0) but improves in neutral-to-basic conditions (pH ∼ 8.0). Nanofibers from which the Ru-complexes were removed from the surface were also used under the same conditions. To remove the Ru-complexes 2–3 mg of nanofibers were suspended twice in an excess of MeCN (20 mL) to dissolve the respective Ru-complexes from the surface. The nanofibers were then employed in HER. These nanofibers did not show any catalytic activity, strengthening the hypothesis that only molecules on the surface are active. SEM images of all three Ru-complex containing fibers after catalytic conditions were applied did not show any changes in the morphology of the fibers (see ESI Figure S2). EDX spectra of all three nanofibers revealed no change in the elemental composition, confirming that the Ru-complexes and associated elements remain incorporated within the PAN matrix under catalytic conditions (see ESI Table S5–7). In contrast to EDX, which probes the whole fiber and integrating over many fibers, with XPS analysis we were able to probe the surface of the fibers with an information depth of 10 nm. For the most part, the XPS data (see ESI Table S1–3) also show no alteration of the molecular composition, with an exception for P, F and Cl. A clear depletion of these elements is observed after catalysis, which is atributed to the dissolution of PF_6_^–^ from all fibers in water and of Cl^–^ from the RutpphzRhCp* containing fibers. Complementary also optical spectroscopy was applied with diffuse reflectance probing the solid fibers, while with UV–vis the fibers dissolved in DMF were analyzed. Diffuse reflectance spectra measured after catalysis, with respect to the solution spectrum in MeCN, exhibited only minor broadening for the nanofibers containing RutpphzPtI_2_ and RutpphzRhCp* and are essentially identical for the nanofibers containing Rutpphz (see ESI Figure S13). Minor changes may be attributed to the slightly different chemical environment of the chromophores in the fiber vs. in solution and the dissolution of PF_6_^–^ and Cl^–^ from the surface as observed with XPS. UV–vis spectra of the fibers, after catalytical conditions were applied, were dissolved in DMF. The received spectra of all three fibers with embedded Ru-complexes are essentially identical to the solution spectra of the respective Ru-complexes in DMF (see ESI Figure S13). In addition, steady-state UV–Vis absorption, emission, and femtosecond transient absorption (fsTA) behavior of the fibers were also recorded before and after catalysis (Figures S14 and S15), which clearly demonstrate the morphological integrity of the fibers and the molecular stability of the embedded Ru-complexes. The spectral features and overall behavior remain unchanged, indicating that the excited-state dynamics of the embedded Ru-complexes is preserved in the fibers before and after catalysis.

For Rutpphz, the lowest activity was observed, as expected since no catalytic center is introduced. This minor activity of Rutpphz for the HER is consistent with earlier findings by Sawaki et al.^59^ who demonstrated HER activity in similar Rutpphz-based systems. The catalytic activity toward proton reduction is enhanced by the introduction of the catalytic centers. Both RutpphzPtI_2_ and RutpphzRhCp* exhibit overall low TONs not reaching those of comparable systems in catalytically optimized homogeneous solutions. In MeCN/water/TEA, RutpphzPtI_2_ achieved a TON of 520 and RutpphzRhCp* a TON of 17.^18,19^ After the first cycle, all three fibers still showed activity toward H_2_ production. The Ru-tpphz fiber after 3 cycles (in total 72 h irradiation) did not give quantifiable amounts of H_2_. Both the RutpphzPtI_2_ and Ru-tpphzRhCp* fibers exhibited a significant drop in H_2_ evolution. After the third cycle (in total 72 h irradiation), the RutpphzPtI_2_ fibers gave a TON of 4, and the Ru-tpphzRhCp* fibers a TON of 2 (see ESI Table S9). From all the collected analytical data (XPS, EDX, UV–vis, emission, diffuse reflectance), we cannot deduce major changes in the composition of the fibers or changes in the chromophore. The loss of PF_6_^–^ is unlikely to influence the catalytic performance as previously the independence of the anion and the catalytic performance was shown.^54,60^ Loss of the Cl^–^ ligand is an essential step in the catalytic cycle^53,61^ and is therefore also not expected to inhibit the catalytic activity. Additionally, the deactivation of the bridge from tpphz to tpphzH_2_,^19^ previously shown to inhibit the catalysis, is not observed in our experiments.

Homogeneous hydrogen catalysis was performed for the Ru-complexes using mainly aqueous sodium ascorbate solution as solvent with the remaining conditions identical to the heterogeneous hydrogen catalysis. To 2 mL of sodium ascorbate (100 mM), 100 μL of the respective Ru-complex dissolved in MeCN were added (see Table S11). The addition of a small amount of MeCN was necessary to ensure the dissolution of the Ru-complexes. Irradiation was performed equally to the experiments above. Control experiments were performed without irradiation, yielding no hydrogen. Reactions under homogeneous conditions gave comparable results to the heterogeneous reactions. For Rutpphz, detectable amounts of H_2_ were found. For RutpphzPtI_2_ a TON of 7, and for RutpphzRhCp*, a TON of 3 was measured. Both are similar to the amounts received by the nanofibers, within the margin of error. Thus, the immobilization of these Ru-complexes has no significant negative impact on catalytic activity. The catalytic solutions were further analyzed by monitoring the reaction with UV–vis spectroscopy. Catalytic reactions were performed inside a glovebox using a cuvette, ensuring the exclusion of air. UV–vis spectra were taken at different reaction times (see ESI Figure S16). In contrast to previous reports using TEA as a sacrificial donor,^18,19^ the formation of the respective reduced tpphzH_2_ complex was not observed in aqueous sodium ascorbate solution. For RutpphzRhCp*, the absorption spectra do not exhibit any significant changes after 20 h of irradiation (see ESI Figure S16) in unison with the high stability proposed in the literature.^18^ For Rutpphz and RutpphzPtI_2_, a decrease and a hypsochromic shift of the visible absorption were observed (see ESI Figure S16). These are in contrast with the diffuse reflectance spectra, which show a minor broadening of the visible absorption without a shift, after catalytic conditions were applied. These differences may arise from the altered chemical environment of the Ru-complexes in homogeneous solution relative to that within the PAN-based nanofiber matrix.

Altogether, these results highlight the significant decrease in catalytic efficiency when transitioning from optimized organic to fully aqueous media. Nevertheless, the aqueous systems, particularly those operating heterogeneously, offer distinct and practically relevant advantages. They operate entirely in a fully aqueous system without the need for organic solvents to aid solubility and employ cheap and ecologically benign sacrificial donors such as sodium ascorbate and ascorbic acid. Moreover, the molecular Ru-based assemblies remain stably immobilized within the recyclable soft-matter matrix, maintaining activity comparable to that observed in the homogeneous aqueous systems while preventing leaching or deactivation.

Heterogeneous hydrogen evolution was further explored in a custom-built membrane flow reactor, fabricated in house from aluminum and Teflon. The windows used for irradiation were made from polyfluoroethylenepropylene (FEP, more information in ESI). For catalytic experiments, an approximately 5 × 5 cm piece of the respective nanofibers was prepared by removing the aluminum collector foil and transferring the nanofibers onto an equally sized piece of FEP, serving as an inert support. The assembled reactor was purged with Ar for 15 min before being filled with 50 mL of 100 mM aqueous sodium ascorbate solution, predegassed with Ar for 2 h. Reactions were performed under continuous flow (20 mL h^–1^) and irradiated for 24 h at 450 nm (100 mW cm^– 2^). TONs were corrected by the amount of leached Ru-complex, in the same way as for the heterogeneous experiments in the vials. Control experiments were performed with pure PAN nanofibers, giving no hydrogen.

All three Ru-complex-containing nanofibers produced detectable amounts of H_2_. For the Rutpphz nanofibers the amount was below the calibration of the GC. The RutpphzPtI_2_ nanofibers gave a TON of 7, and the RutpphzRhCp* nanofibers a TON of 1. The values achieved for the flow reactor are similar to the values achieved in the vial (batch reactor). These results initially show the feasibility of transferring small-scale batch catalytic experiments to flow conditions aimed to be scaled up in the future while maintaining catalytic activity. The similar TONs in the membrane reactor showcase the gastight design of the reactor, comparable to the commonly used vial reactors, maintaining an inert atmosphere over the whole irradiation time. Conditions were not optimized yet and only serve as a proof of concept for possible pathways to scale up HER to an industrial scale.

## Conclusion

In this report, we could show how Ru-complexes can be easily embedded into a PAN soft matter by electrospinning. XPS, EDX, NMR, UV–vis, diffuse reflectance, FT-Raman, and ATR-IR verified the experimental composition to match the theoretical one, while O-PTIR mapping of the nanofibers showed a homogeneous distribution of the embedded Ru-complexes within the nanofibers. The nanofibrous material is capable of performing HER in aqueous media with both sodium ascorbate and ascorbic acid as sacrificial donors. Sodium ascorbate gave overall better results, yielding TONs of 3 (RutpphzPtI_2_) and 1 (RutpphzRhCp*). The catalytic performance is lower than literature reports in optimized organic media but is similar to homogeneous catalysis in mostly aqueous solutions. Additionally, the transfer of the small-scale vial reaction to a membrane flow reactor was successful, giving similar results as the vial reactions.

The steady-state absorption study of these molecular PMDs in nanofibers show a red shift in the absorption maxima, accompanied by spectral broadening attributed to aggregation and alterations of the local environment by the PAN matrix. Emission measurements in a polymeric solution of DMF indicate that the nitrile interacts strongly with the Pt center, whereas the sterically bulky RhCp* unit remains largely unaffected. In the nanofibers, the emission of the molecular PMDs is blue-shifted due to reduced stabilization of the π* orbitals of tpphz, with the effect being most pronounced in RutpphzRhCp*. The electron transfer rate across the tpphz bridge is observed to be 24 times slower than previously reported for these complexes, suggesting higher barriers for intramolecular electron transfer resulting from restricted mobility inside the fibers. Residual emission from the dinuclear complexes hinted toward buried complexes with suboptimal alignment that do not favor electron transfer. TA measurements revealed that, irrespective of metal coordination, the ILCT occurs on a subpicosecond time scale in the nanofibers. In the mononuclear complex, the population density is delocalized over the entire tpphz ligand, undergoing radiative decay on the submicrosecond time scale, whereas in the dinuclear complexes, the density is transferred to the catalytic center. This transfer occurs 8.4 times slower in RutpphzRhCp* than in RutpphzPtI_2_, likely highlighting the larger geometric changes that have to occur upon reduction of the Rh center. The electron transfer time obtained from fsTA for the dyads indicated coexistence of surface and buried molecules, wherein the surface molecules enable a faster electron transfer across the bridge and are likely responsible for the observed catalytic activity. These findings provide mechanistic insight for the rational design of fiber-supported PMD-based heterogeneous catalytic systems.

## Experimental Section/Methods

### Materials

All chemicals (purities ≥ 99%) were purchased from Sigma-Aldrich Chemie GmbH (Munich, Germany). Electrospinning equipment/consumables were purchased from Fluidnatek (e.g., needle, syringe adapter). Tubes used in electrospinning were purchased from Bola, and aluminum foil was purchased from VWR Avantor.

### Electrospinning Conditions

The electrospun nanofibers were prepared using a Fluidnatek LE-50 instrument operated in a horizontal setup. A 14-gauge needle was used and aluminum foil-covered collector. Electrospinning parameters include a needle-to-collector distance of 15 cm, an applied voltage of 13 kV, and a flow rate of 500 μL h^–1^. Spinning solution (total mass of 5 g) consisting of 0.25 g of PAN and 0.031 g of the respective Ru-complex dissolved in dimethylformamide (DMF), was stirred for 48 h. The resulting homogeneous solution was transferred into a syringe and electrospun directly onto aluminum foil.

### Characterization

SEM images were acquired on a Thermo Fisher Apreo2 instrument at an acceleration voltage of 5 kV (spot size: 0.4 nA). Beam deacceleration was applied to improve the image quality by using a stage bias of 600 V. XPS measurements were performed on a Kratos Ultra DLD with monochromatic Al Kα radiation, referencing spectra to C 1s at 284.7 eV. Elemental analysis was carried out by EDX using a Zeiss Gemini Ultra 55 SEM with a Bruker XFlash detector. Additional vibrational characterization was performed by ATR-IR, FT-Raman, and O-PTIR spectroscopy.

### Optical Spectroscopy

UV–vis absorption spectra were recorded on a JASCO V-780 spectrometer, and emission spectra were recorded on an Edinburgh Instruments FLS980 spectrophotometer, collected in reflection mode from samples in a quartz cell (10 mm path length). Diffuse reflectance spectra were taken on a UV-2600 UV–vis spectrophotometer (Shimadzu, Japan) equipped with an integrating sphere. Spectra were taken against a BaSO_4_ standard as a baseline and analyzed via the Kubelka–Munk approach.

### Transient Absorption Spectroscopy

Femtosecond and nanosecond transient absorption spectra were captured by using a pump–probe setup. The sample was excited by a 400 nm pump beam generated from a BBO. The data were collected in UV–vis region (350 nm – 700 nm) using a white light probe generated by self-phase modulation of an 800 nm beam in a rotating CaF_2_ crystal.

### Photocatalysis Conditions

Light-driven hydrogen evolution experiments were conducted in aqueous media using environmentally benign sacrificial donors such as sodium ascorbate and ascorbic acid. Samples were illuminated at 450 nm with P = 0.08 W/cm^2^, under rigorous ventilation to avoid the buildup of heat. Hydrogen was quantified by gas chromatography (GC). Leaching and flow reactor experiments are described in the Supporting Information.

## Supplementary Material



## Notes

Additional experimental details, materials, fiber preparation, SEM images, XPS, and EDX analysis of Rutpphz, RutpphzPtI_2_ and RutpphzRhCp* fibers before and after catalysis. Vibrational spectroscopy (FT-Raman spectroscopy, ATR-IR, diffuse reflectance), steady-state absorption and emission spectroscopy, and UV–vis analysis of Rutpphz, RutpphzPtI_2_ and RutpphzRhCp*. Leaching experiments, postcatalysis steady-state and transient spectra (fsTA, nsTA), gas chromatography (GC) measurements for hydrogen quantification, UV–vis analysis of the Ru-complexes before and after 24 h irradiation with 450 nm under homogeneous catalytic conditions, schematic representation of the membrane reactor, ^19^F NMR spectrum (376 MHz) of Rutpphz, RutpphzPtI_2_ and RutpphzRhCp* fibers, and nsTA time-dependent spectra and kinetics of these fibers (PDF)

## ○

Institute for Fiber Engineering and Science (IFES), Research Cluster for Social Implementation (RCSI), Shinshu University, Tokida 3–15–1, Ueda, Nagano 386–8567, Japan

## Abbreviations

PMD: Photochemical molecular device
PAN: polyacrylonitrile
FEP: polyfluoroethylenepropylene
UV–vis: ultraviolet–visible
IR: infrared
ATRIR: attenuated total reflection infrared
FT: Fourier transform
O-PTIR: optical photothermal infrared
NMR: nuclear magnetic resonance
