# Supplementary material for: Soft-Matter Confinement Modulates Excited-State Dynamics of Ru Photocatalysts for Hydrogen Evolution in Aqueous Media
Source: ACS Mater Au. 2026 Feb 23;6(3):553–65. doi: 10.1021/acsmaterialsau.5c00212 (PMC13177401; doi:10.1021/acsmaterialsau.5c00212)
Supplement: Supplementary file 1 [file mg5c00212_si_001.pdf]

Supporting Information

Soft-matter confinement modulates excited-state dynamics of Ru-photocatalysts for hydrogen evolution in aqueous media

Jens H. Tran[+,a], Nikita Vashistha,[+,b], Akuila Edwards[b,c], Alexander K. Mengele[a], Alina Koba[d], Sana Ullah[a†], Jens Bauer[e], Jan Griebel[e], Michael Schmitt[b,f], Samir F. El-Mashtoly[b], Jürgen Popp[b,c]*, Dirk Ziegenbalg[d]*, Benjamin Dietzek-Ivanšić[e,]*, Sven Rau[a,]*, Montaha Anjass[a,g,]*.

* Corresponding authors

\+ These authors contributed equally to this work.

a) Institute of Inorganic Chemistry I, Ulm University, Albert-Einstein-Allee 11, 89081 Ulm, Germany.

E-Mail: montaha.anjass@uni-ulm.de, sven.rau@uni-ulm.de

b) Leibniz Institute of Photonic Technology, e. V. Jena, Albert-Einstein-Straße 9, 07745 Jena, Germany.

E-Mail: juergen.popp@leibniz-ipht.de

c) Institute of Physical Chemistry, Friedrich-Schiller-University Jena, Helmholzweg 4, 07743 Jena, Germany.

E-Mail: juergen.popp@uni-jena.de

d) Institute of Chemical Engineering, Ulm University, Albert-Einstein-Allee 11, 89081 Ulm, Germany

E-mail: dirk.ziegenbalg@uni-ulm.de

e) Leibniz Institute of Surface Engineering (IOM), Permoserstraße 15, 04318 Leipzig, Germany.

E-Mail: benjamin.dietzek-ivansic@iom-leipzig.de

f) Abbe Center of Photonics, Albert-Einstein-Straße 6, 07745 Jena, Germany.

g) Department of Chemistry, University of Sharjah, 27272 Sharjah, United Arab Emirates

E-Mail: malmasri@sharjah.ac.ae

General remarks

Materials: All used chemicals were purchased from Sigma-Aldrich Chemie GmbH (Münich, Germany) with purities of 99 % and higher. Electrospinning equipment was purchased from Fluidnatek (needle, syringe adapter etc.). Tubes used in electrospinning were purchased from Bola and aluminum foil from VWR Avantor. Synthesis of the Ru-complexes were published previously.[1,2]

Fiber Preparation: The electrospun nanofibers were fabricated using a Fluidnatek, LE-50 instrument operated in a horizontal setup. A 14-gauge needle was used, and the collector was covered in aluminum foil. The applied electrospinning parameters includes a needle-to-collector distance of 15 cm, an applied voltage of 13 kV, and a flow rate of 500 µL h^{-1}. For solution preparation, a total mass of 5 g was prepared. Consisting of 0.25 g of PAN (Sigma-Aldrich, M_m= 150,000 g mol^{-1}, 10 wt% relative to the total solution), and 0.031 g of the respective Ru-complex (0.625 wt% with respect of the total solution). The components were dissolved in dimethylformamide (DMF), stirred for 48 hours, and the resulting homogeneous solution was transferred into a syringe and electrospun directly onto aluminum foil. The obtained nanofiber mats exhibited orange color, with a theoretical loading of 5.9 wt% of the respective Ru-complex.

SEM images

Measurements were performed on a Thermo Fischer Apreo2 device placed on an anti-vibrational table and surrounded by a magnetic shield to avoid noise. Images were acquired at an acceleration voltage of 5 kV and spot size of 0.4 nA. Beam deacceleration was applied to improve the image quality by using a stage bias of 600 V. Nanofiber samples were mechanically detached from the aluminum foil and secured with carbon tape (Plano) onto aluminum stubs.

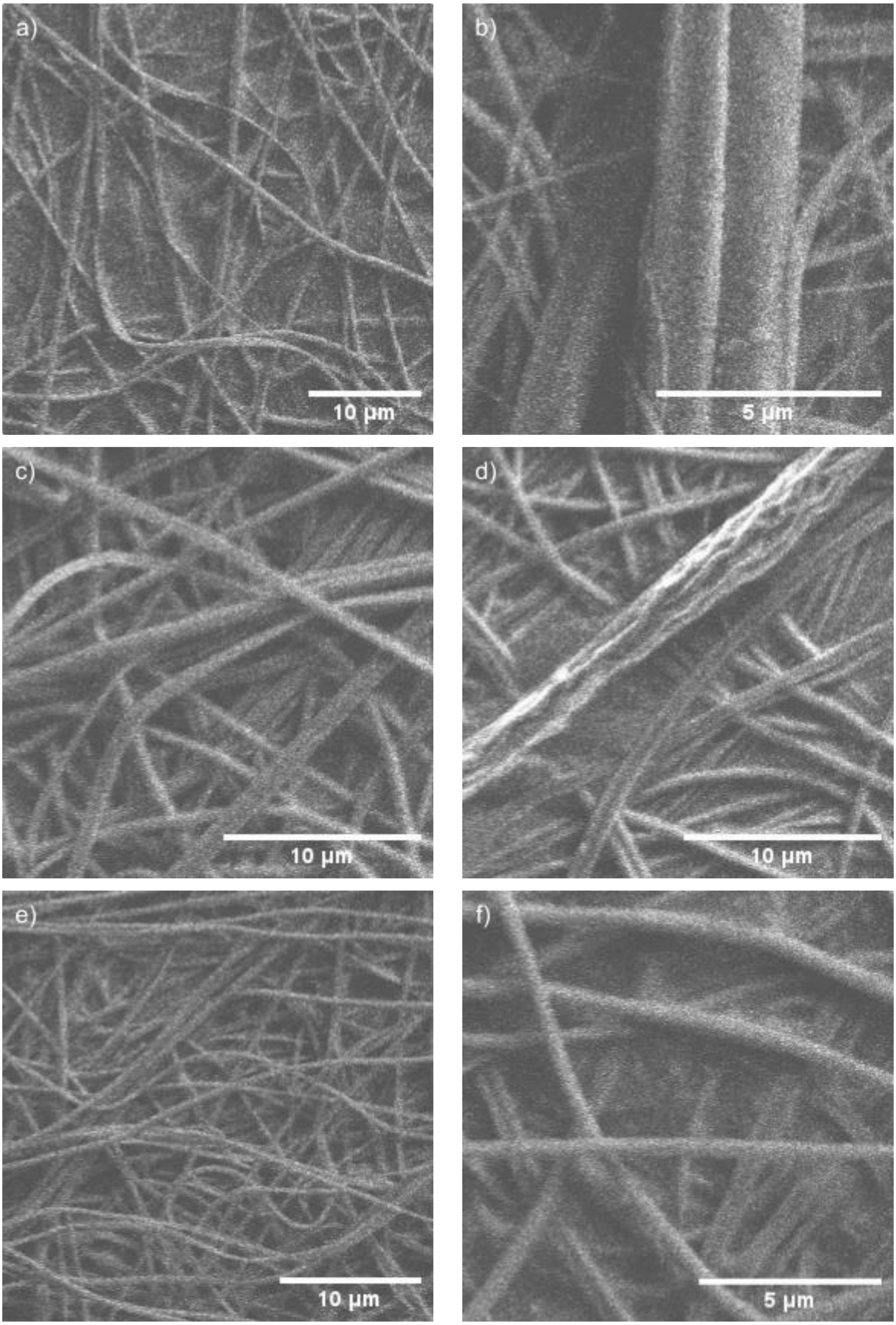

Figure S1: SEM images of Rutpphz fibers (a, b), $RutpphzPtI_2$ fibers (c, d) and RutpphzRhCp* fibers (e, f), before catalysis.

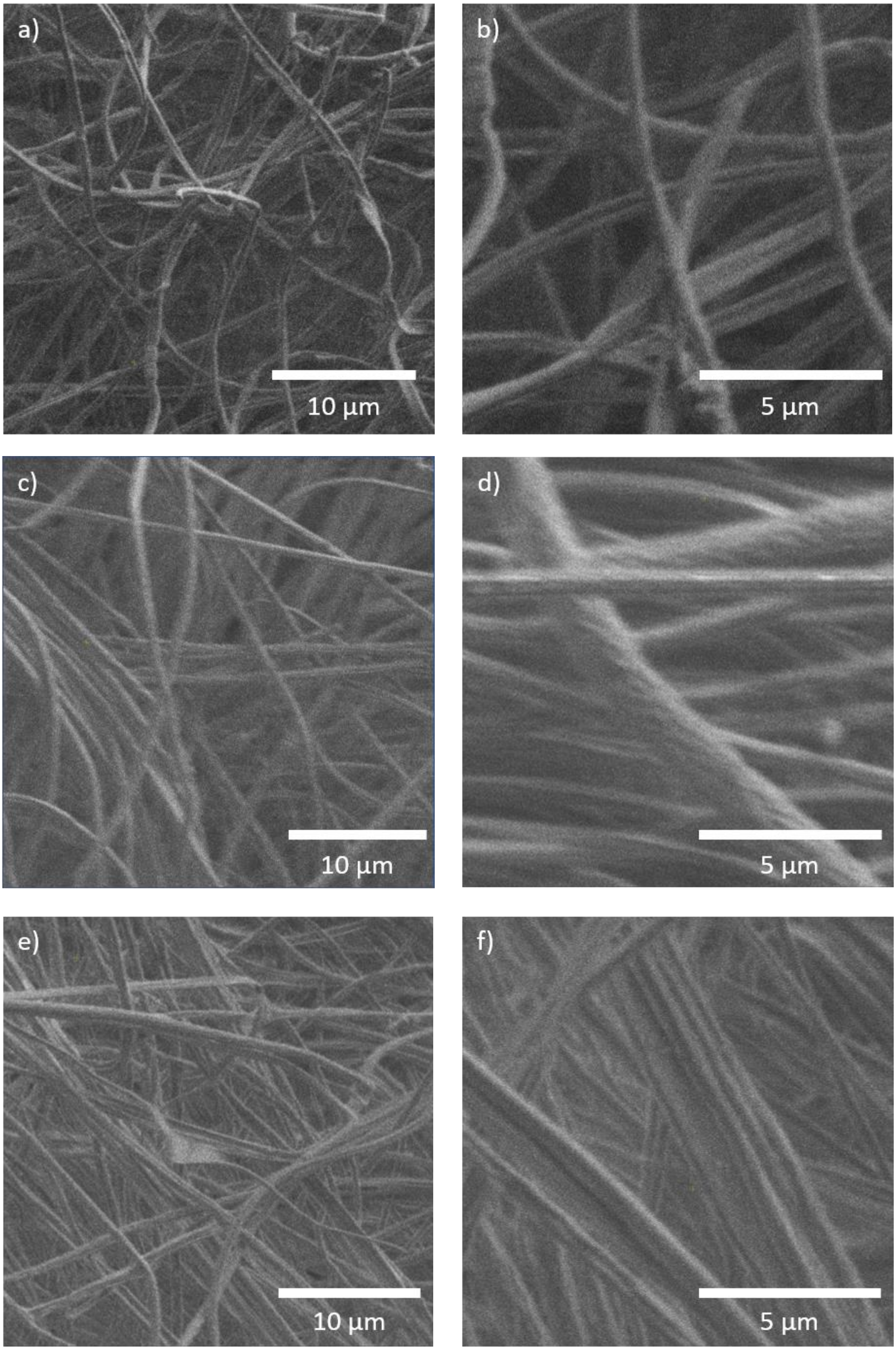

Figure S 2: SEM images of Rutpphz fibers (a, b), $RutpphzPtI_2$ fibers (c, d) and RutpphzRhCp* fibers (e, f) after catalysis.

XPS measurements[3–5]

The surface composition is explored by X-ray photoelectron spectroscopy (XPS, Kratos Ultra DLD). The measurements are performed at room temperature with a background pressure of 1 x 10^{-7} Pa. For excitation, monochromic Al K_α radiation at 1486.6 eV is used. The X-ray source is operated at 150 W. Photoelectrons are released from the ~220 µm analysis area and are analysed under normal incidence configuration. The photoelectron yield is enhanced applying a magnetic immersion lens. To avoid sample charging a neutralizer supplies thermal electrons to the analysis area. A retarding lens system coupled by a hemispherical analyser with 40 eV pass energy is used for energy separation. The energy-filtered photoelectrons are amplified passing a microchannel plate and recorded by a delay line detector. The UNIFIT 2025 software is used for spectra analysis and composition calculation considering the specific transmission correction of the XPS machine. For curve fitting convolved Gaussian-Lorentzian peak profiles are simultaneously optimized with a Shirley background profile. The spectral calibration is set to the aromatic sp^2 carbon peak in the C 1s core-level spectrum at 284.7 eV.

Table S1*:* Elemental composition determined by XPS for Rutpphz fibers.

Element	Theoretical value / wt.%	Experimental value before catalysis / wt.%	Experimental value after catalysis / wt.%
C	71.15	70.93	72.24
F	1.09	3.42	0.70
O	0.00	2.44	3.95
N	26.99	19.90	21.58
P	0.30	0.60	0.10
Ru	0.48	2.11	1.43

Table S2: Elemental composition determined by XPS for $RutpphzPtI_2$ fibers.

Element	Theoretical value / wt.%	Experimental value before catalysis / wt.%	Experimental value after catalysis / wt.%
C	70.22	61.53	68.09
F	0.81	1.39	0.50
O	0.00	10.82	8.47
N	26.80	18.20	20.90
P	0.22	1.08	0.00
Ru	0.36	0.81	0.69
Pt	0.69	4.24	1.92
I	0.90	1.93	1.63

Table S3: Elemental composition determined by XPS for RutpphzRhCp* fibers

Element	Theoretical value / wt.%	Experimental value before catalysis / wt.%	Experimental value after catalysis / wt.%
C	70.96	68.07	68.94
F	0.88	3.33	0.26
O	0.00	2.03	4.55
N	26.86	21.38	22.48
P	0.24	0.59	0
Ru	0.39	1.22	1.09
Rh	0.40	3.19	2.69
Cl	0.27	0.19	0

Peak fitting and assignment were performed using the UNIFIT2025 database and NIST (https://srdata.nist.gov/xps/). F 1s was fitted by [4] and by [5]. The lower spectrum shows the XPS before and the upper after catalysis.

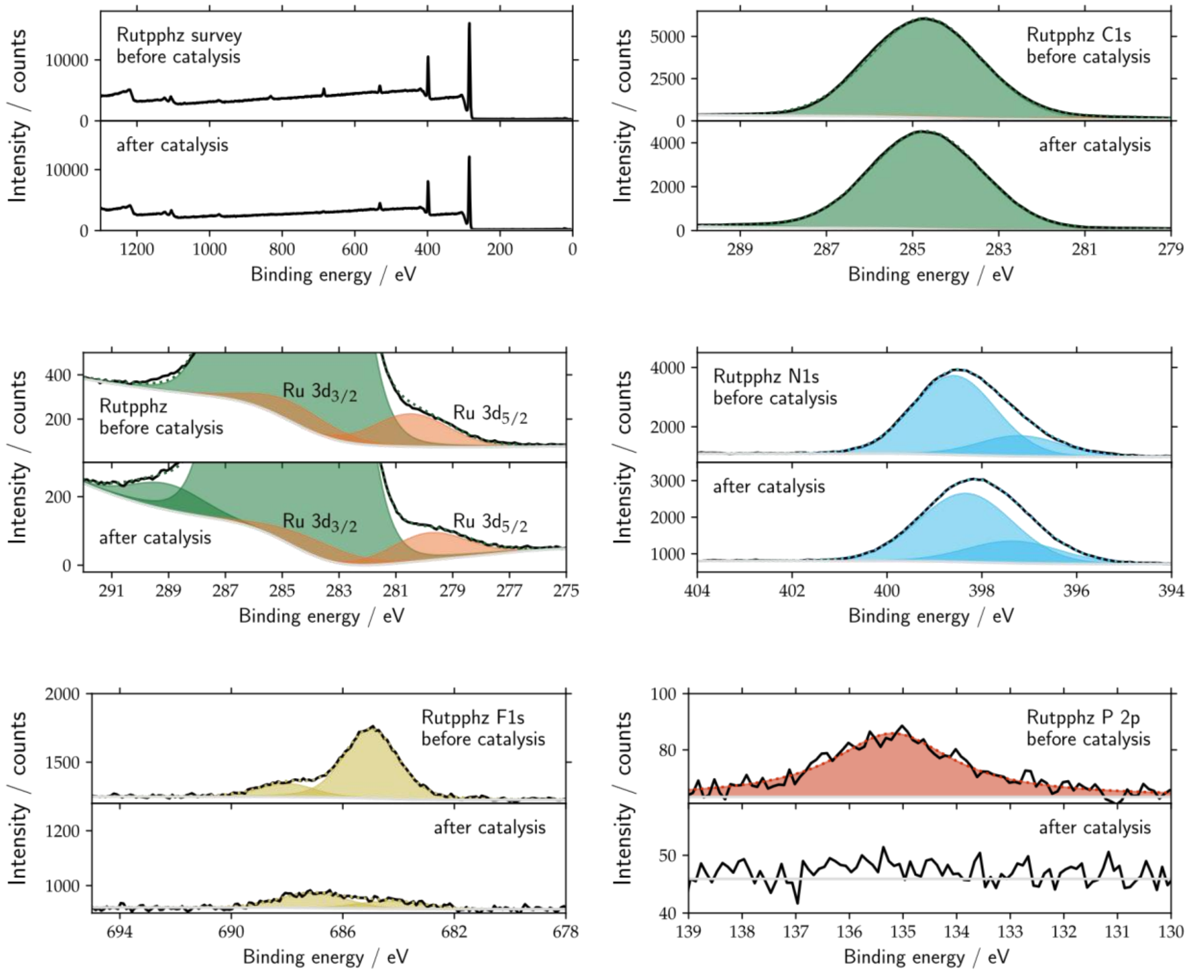

Figure S3: XPS spectra for Rutpphz fibers. Upper spectrum is before catalysis and lower after catalysis.

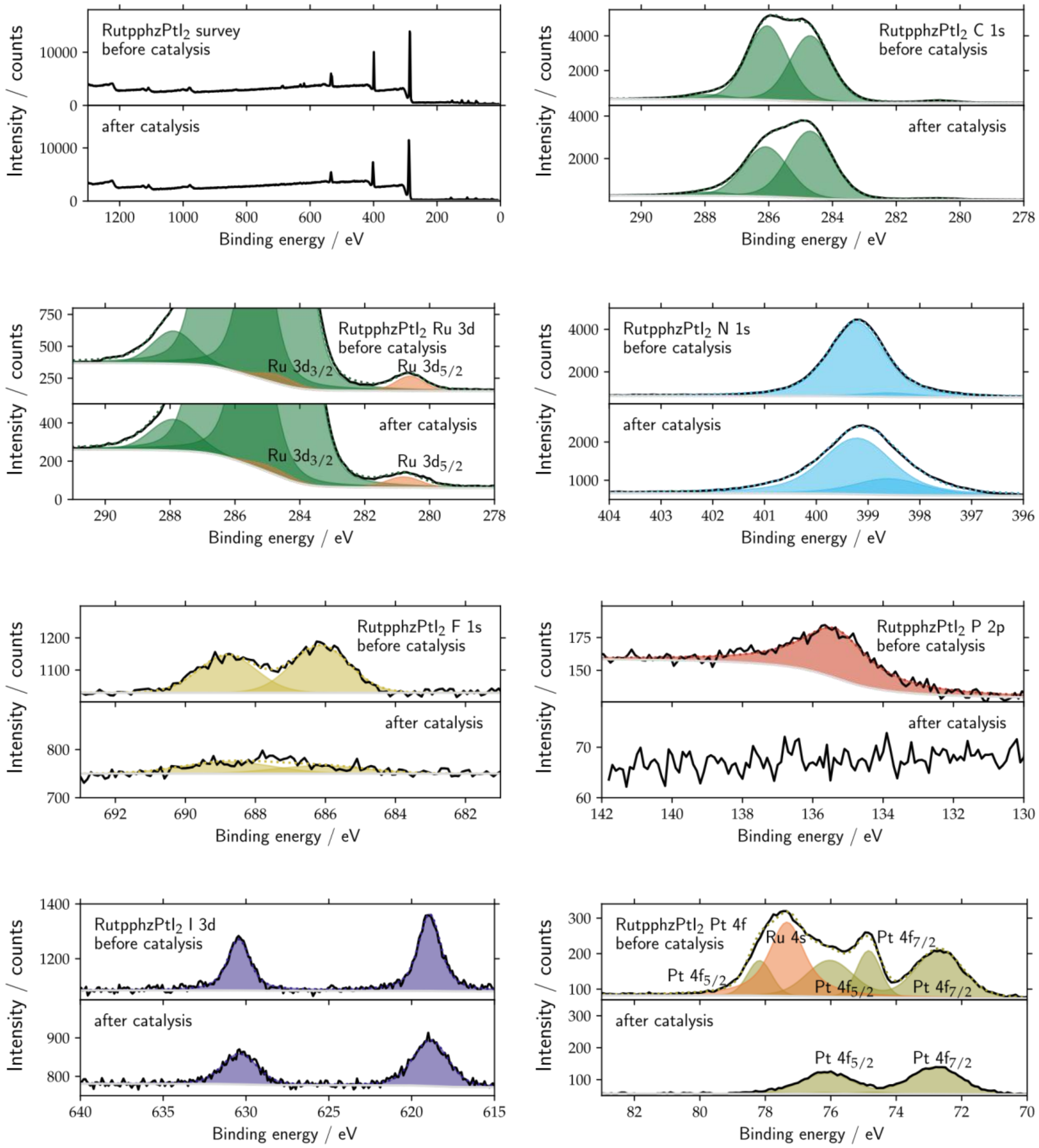

Figure S 4: XPS spectra for RutpphzPtI$_2$ fibers. Upper spectrum is before catalysis and lower after catalysis.

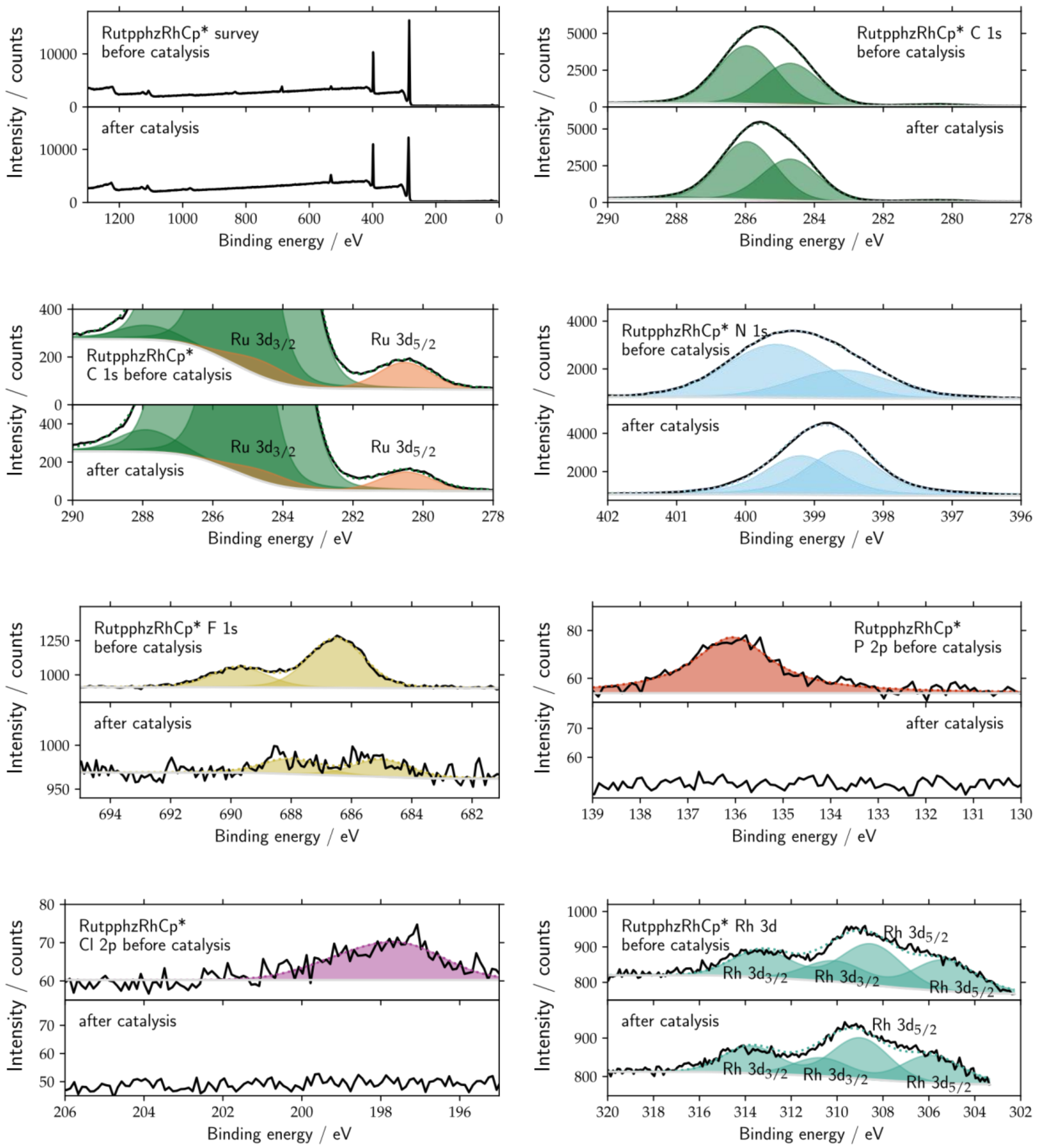

Figure S5: XPS spectra for RutpphzRhCp* fibers. Upper spectrum is before catalysis and lower after catalysis.

EDX measurements[6]

EDX spectra were measured in a Zeiss Gemini Ultra 55 SEM with a Bruker XFlash 6–30 detector. A beam voltage of 5 kV, beam aperture of 30 µm, and a working distance of 5.3 mm were applied. Applying 5 kV achieved a good spatial resolution, despite the low voltage fibers were probed as a whole. The structures moved under the electron beam due to local charging. To increase the accuracy of the determined composition EDX was accumulated over a bunch of fibers on an area of 38.6 µm × 29.0 µm

for 14 hours. Series deconvolution including the SEM background fitting was applied for spectra evaluation. For composition analysis the K-lines were used for C, O, F, N, P, Cl, the L-lines for Ru and Rh, and the M-lines for Pt and I. The measurement errors were calculated by the fitting error (two sigma accuracy) combined with an absolute systematic error of 0.1 wt%.

Table S4: Elemental composition determined by EDX for pure PAN fibers.

Element	Theoretical value / wt.%	Experimental value before catalysis / wt.%
C	65.91	81.4±4.4
O	0.00	2.19±0.25
N	34.09	16.4±1.4

Table S5: Elemental composition determined by EDX for Rutpphz fibers.

Element	Theoretical value / wt.%	Experimental value before catalysis / wt.%	Experimental value after catalysis / wt.%
C	71.15	81.9 ± 4.4	82.6 ± 4.5
F	1.09	0.37 ± 0.12	0.29 ± 0.12
O	0.00	2.11 ± 0.24	1.69 ± 0.21
N	26.99	14.2 ± 1.2	14.2 ± 1.2
P	0.30	0.44 ± 0.13	0.32 ± 0.12
Ru	0.48	0.94 ± 0.17	0.87 ± 0.17

Table S6: Elemental composition determined by EDX for $RutpphzPtI_2$ fibers.

Element	Theoretical value / wt.%	Experimental value before catalysis / wt.%	Experimental value after catalysis / wt.%
C	70.22	80.2 ± 4.3	82.8 ± 4.5
F	0.81	0.19 ± 0.11	0.19 ± 0.11
O	0.00	1.58 ± 0.21	1.38 ± 0.19
N	26.80	14.5 ± 1.2	12.0 ± 1.1
P	0.22	0.16 ± 0.11	0.17 ± 0.11
Ru	0.36	0.52 ± 0.14	0.74 ± 0.16
Pt	0.69	1.24 ± 0.17	1.57 ± 0.19
I	0.90	1.62 ± 0.22	1.11 ± 0.18

Table S7: Elemental composition determined by EDX for RutpphzRhCp* fibers

Element	Theoretical value / wt.%	Experimental value before catalysis / wt.%	Experimental value after catalysis / wt.%
C	70.96	83.0 ± 4.5	76.8 ± 4.2
F	0.88	0.25 ± 0.11	0.42 ± 0.12
O	0.00	1.77 ± 0.22	1.98 ± 0.24
N	26.86	12.6 ± 1.1	18.8 ± 1.6
P	0.24	0.29 ± 0.12	0.31 ± 0.12
Ru	0.39	0.85 ± 0.17	0.69 ± 0.15
Rh	0.40	0.79 ± 0.17	0.73 ± 0.16
Cl	0.27	0.43 ± 0.13	0.19 ± 0.12

Vibrational spectroscopy[2,7,8]

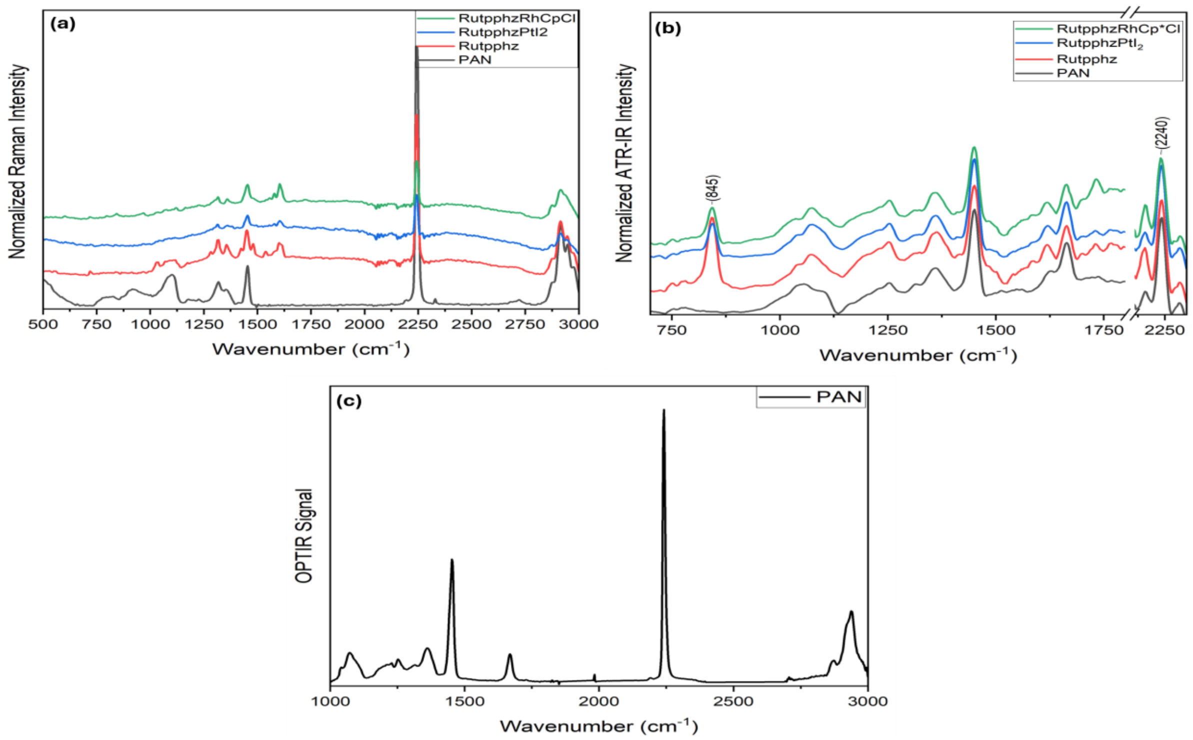

Figure S6: a) Raman spectra of Ru-complex containing PAN nanofibers highlighting vibrations outside the PAN region (>1500 cm^{-1}). Rutpphz-specific modes are observed at 1538 and 1604 cm^{-1}, while Pt- and Rh-containing dyads exhibit a novel peak at 1580 cm^{-1}. The mononuclear dyad shows characteristic shoulders at 1424 and 1480 cm^{-1}. b) ATR-IR spectra of bulk dyads showing the PF_6^- anion vibration at 845 cm^{-1}. c) OPTIR of pure PAN fiber.

FT-Raman spectroscopy provided complementary evidence for the Ru-complex incorporation within the nanofibers. The bulk measurement shows vibrations in the fingerprint region associated with bipyridine groups within the dyad structures. In the mononuclear dyad, shoulders at 1424 and 1480 cm^{-1} were observed, which disappear in the Rutpphz complex, likely due to electronic coupling or charge-transfer interactions. Distinct Rutpphz vibrations corresponding to aromatic C=C and C=N stretching

appeared at 1538 and 1604 cm^{-1}. Additional Raman-active modes included C–N stretching and C–H bending at 1283 cm^{-1}, C–C stretching at 1424 cm^{-1}, and C=C/C=N stretching at 1481 cm^{-1}, all specific to Rutpphz. A novel peak at 1580 cm^{-1} was observed in the Pt- and Rh-containing dyads, appearing as a shoulder in Rutpphz, likely reflecting metal coordination effects on the Rutpphz core. These results are shown in Figure S6 (a).

ATRIR spectroscopy was performed to detect PF_6^- anion vibrations, which lie outside the O-PTIR accessible wavenumber range. Measurements were carried out using a diamond ATR crystal, with the chamber flushed with dry air to remove water vapor. To improve the signal-to-noise ratio and enhance the detection of weak bands, 500 scans were averaged per sample. Data were processed using the in-house Ramanmetrix software. Baseline correction employed the Sensitive Nonlinear Iterative Peak (SNIP) clipping algorithm with 40 iterations. Spectra were area-normalized within nitrile CN stretch, chosen as a stable internal reference. The spectra were then plotted in Origin software and a spectral smoothing window of 10 points was applied. Figure S6 (b) shows the PF_6^- anion vibration observed at 845 cm^{-1}, in agreement with previous FTIR studies,[9,10] and no bands indicative of PF_6^- decomposition were detected, confirming structural integrity during electrospinning. Figure S2(c) shows the OPTIR spectrum of pure PAN fiber showing no additional peaks which belong to the complexes.

Steady-state absorption and emission spectroscopy

Measurements were recorded on a JASCO spectrometer V-780, while an Edinburgh Instruments FLS980 spectrophotometer was used to acquire steady-state emission spectra. Data were collected in reflection mode from a sample placed in a quartz cell. UV-vis spectra recorded inside of the glove box were taken with an Avantes AvaSpec-ULS2048CL detector unit coupled with an AVA AvaLight-DH-S-BAL light source. Light source, cuvette holder and detector were connected with fibreoptic cables. Pathlength of the quartz cells in all cases was 10 mm.

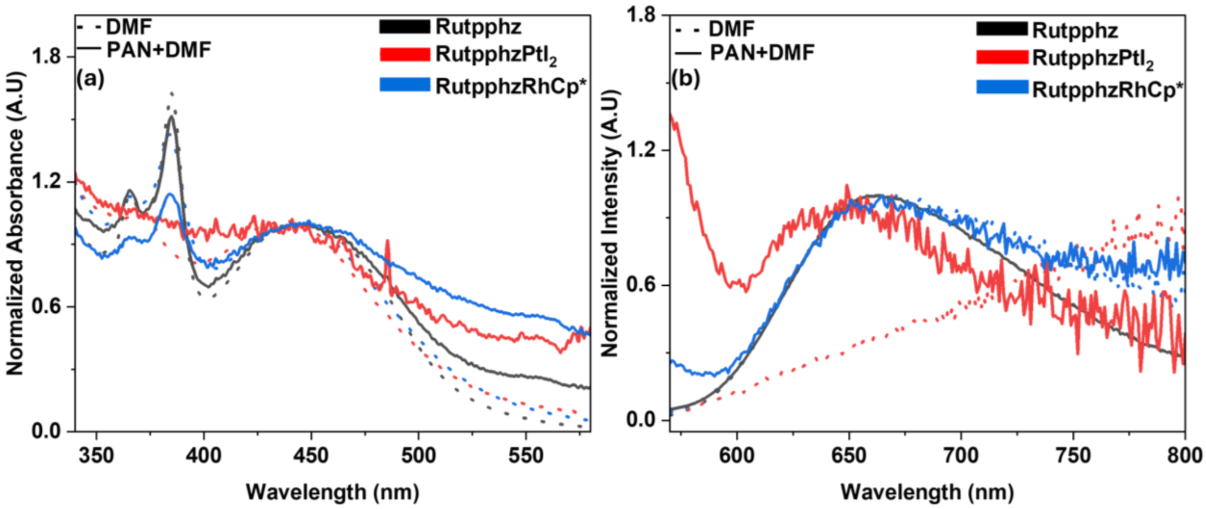

Figure S7: a) Normalized steady state absorption of the Ru-complexes in DMF and the Ru-complexes embedded in PAN nanofibers dissolved in DMF.

Determination of the composition with UV-vis

Composition was determined by taking approximately 1 cm × 1 cm pieces of the fiber sheet and dissolving them in 1.9 g (2 mL) of DMF. The loading with the respective Ru-complexes was determined from the visible absorption maximum. For all three samples: Rutpphz, RutpphzPtI$_2$ and RutpphzRhCp* duplicates were performed. The received loadings agree with the theoretical loading from the fabrication process.

Table S8: Quantitative estimation of photocatalyst loading in 1 cm x 1 cm nanofiber samples.

Fibers	M / g/mol	Total mass fiber / mg	Concentration / µmol/L	Mole / nmol	Mass complex / µg	Concentration Ru complex in the fiber / wt.%
Rutpphz	1312.18	0.650	16.150	32.3	42.4	6.5
RutpphzPtI$_2$	1761.07	0.200	3.712	7.42	13.1	6.5
RutpphzRhCp*	1621.22	0.365	7.420	14.8	24.1	6.6

Leaching experiments

Spectra for the leaching experiments were taken on a V-670 JASCO UV-VIS-NIR Spectrometer. Quartz cuvettes with an optical path length of 1 cm were employed in the measurements. In total five solvents with different polarity were tested. Only in MeCN considerable leaching was observed.

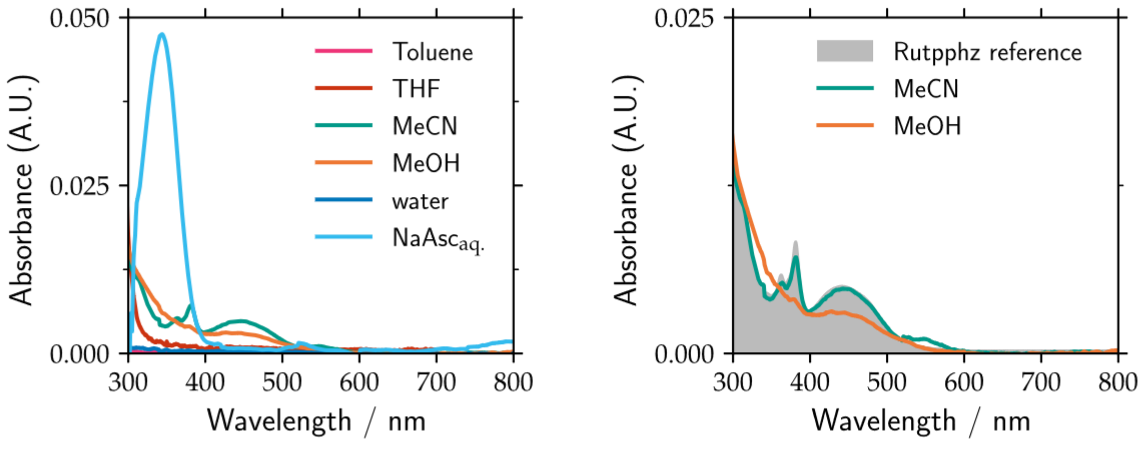

Figure S8: Leaching of Rutpphz fibers in various solvents (left) and zoomed region with solvents in which leaching occurs (right).

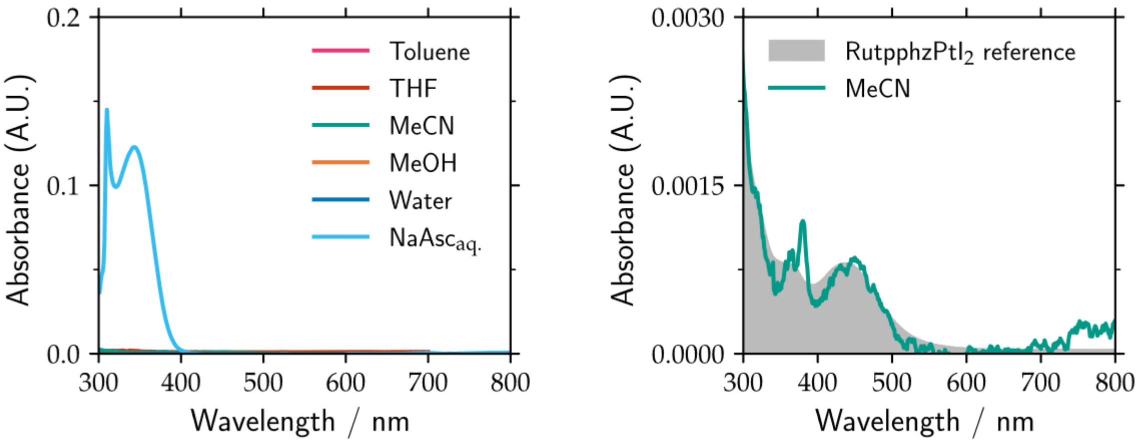

Figure S9: Leaching of $RutpphzPtI_2$ fibers in various solvents (left) and zoomed region with solvents in which leaching occurs (right).

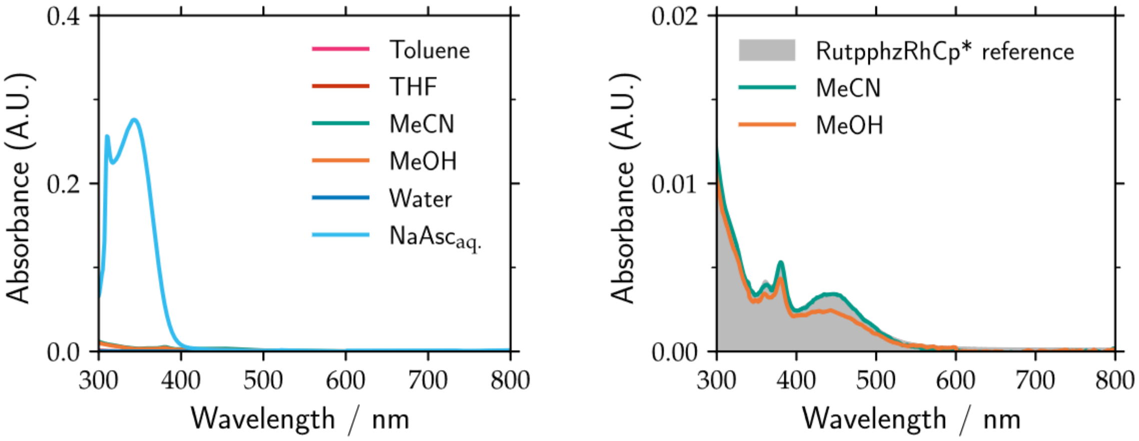

Figure S10: Leaching of $RutpphzPtI_2$ fibers in various solvents (left) and zoomed region with solvents in which leaching occurs (right).

Diffuse reflectance spectra

Diffuse reflectance spectra were taken on a UV-2600 UV-Vis spectrophotometer (Shimadzu, Japan) equipped with the integrating sphere. Spectra were taken against a $BaSO_4$ standard as baseline. Samples were measured directly as received after the electrospinning process on aluminum foil or were placed back onto aluminum foil after catalytic experiments for measurements. Additionally, samples were secured between two microscopy slides. The aluminum foil and microscopy slides were subtracted as a background. Reflectance spectra were transformed using Kubelka-Munk (KM) theory: K-M units = $(1-R)^2/2R$.

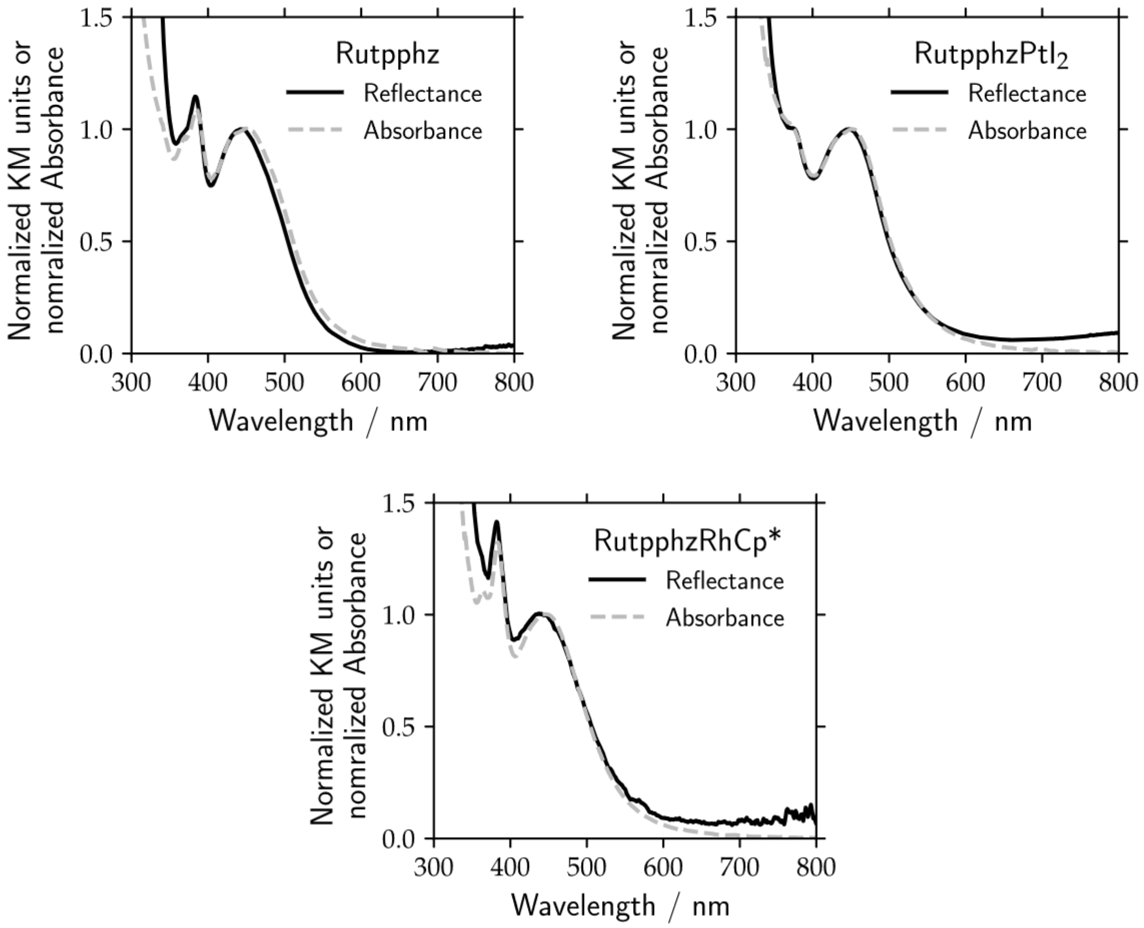

Figure S11: Diffuse reflectance spectra compared with the absorption spectra of the Ru-complexes in MeCN.

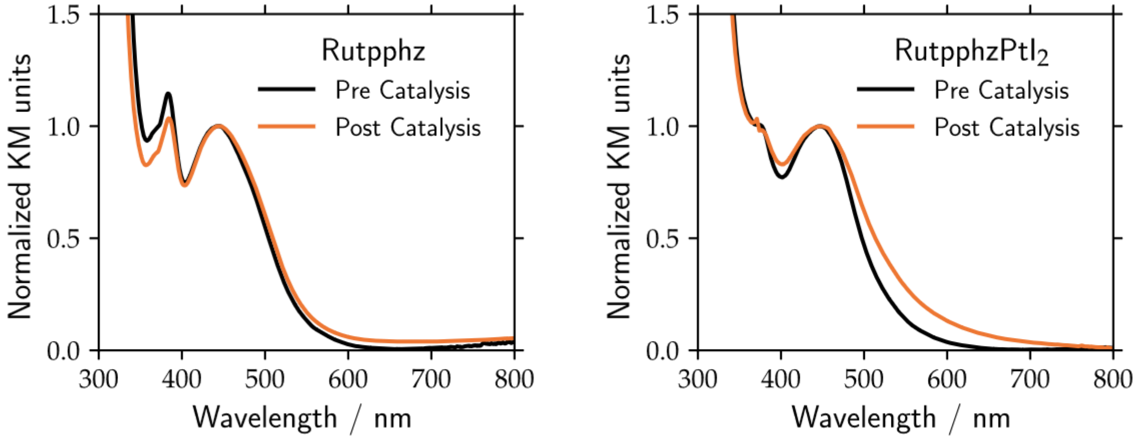

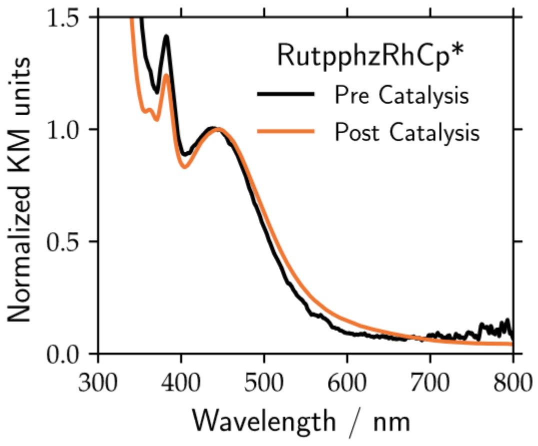

Figure S12: Diffuse reflectance spectra of the Ru-complex fibers before and after catalysis.

Post-catalysis steady state and transient spectra

Between 2-3 mg of post-catalysis fibers were washed twice with 20 mL and dried *in vacuo.* The fibers were then dissolved in DMF.

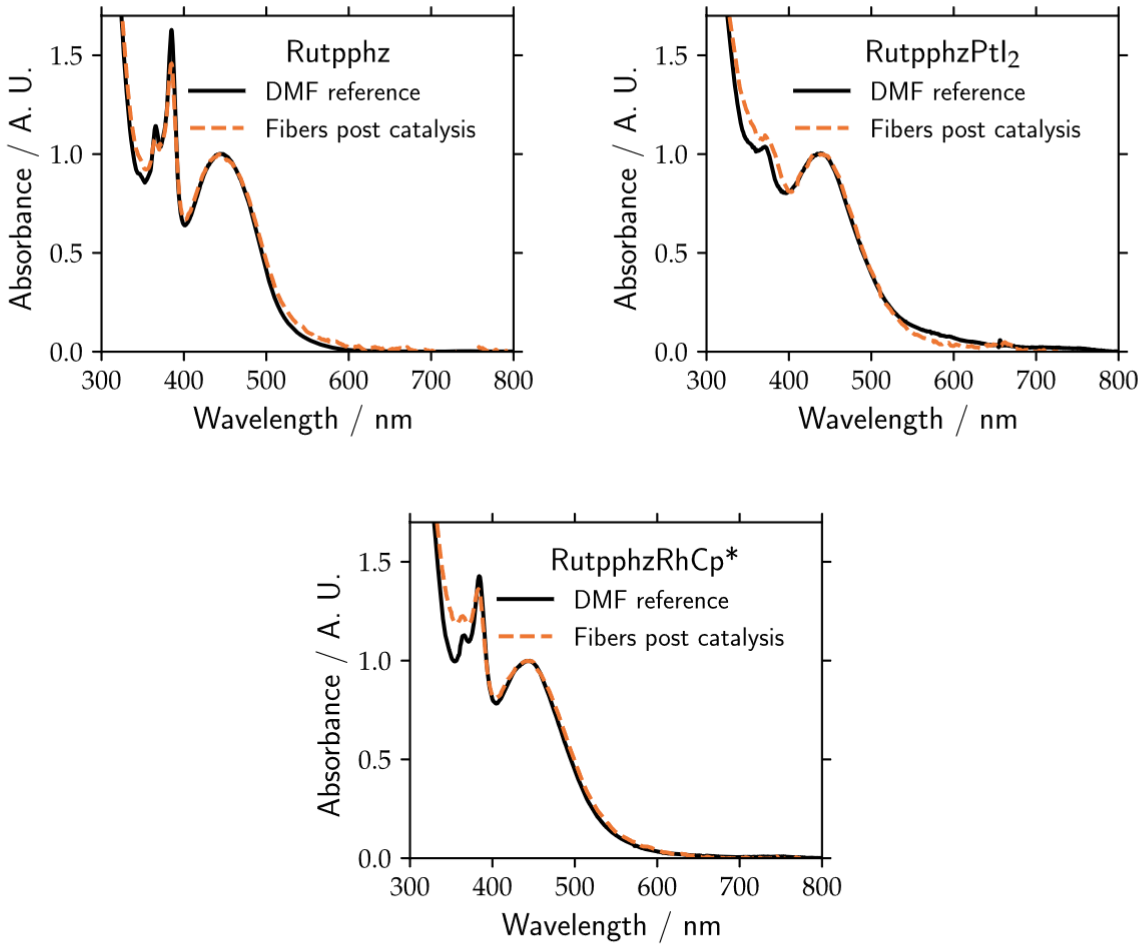

Figure S13: Fibers with embedded Ru-complexes, after catalytic conditions were applied were dissolved in DMF and are compared to the solution spectrum of the native Ru-complexes.

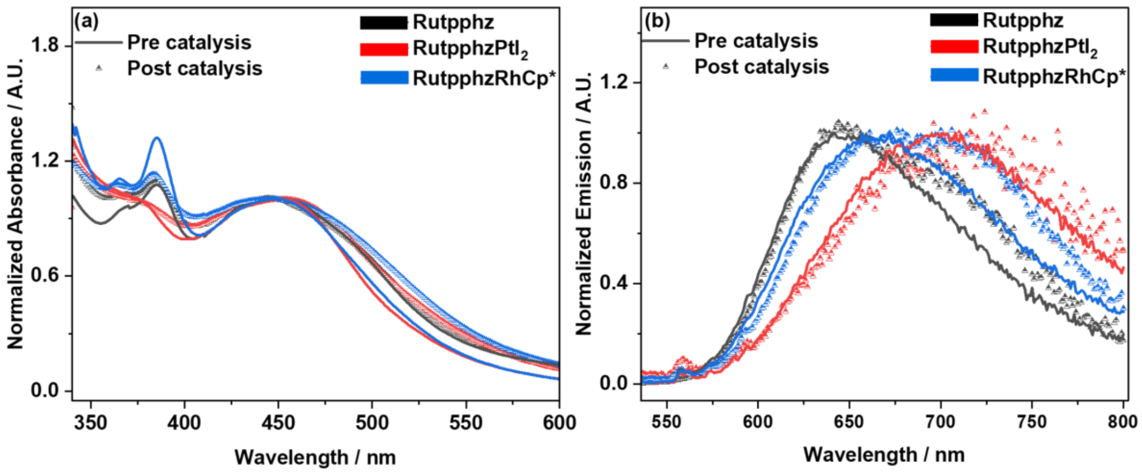

Figure S14: Normalized steady-state a) absorption and b) emission of fibers with embedded Ru-complexes, before (solid line) and after (scatter plot) catalysis.

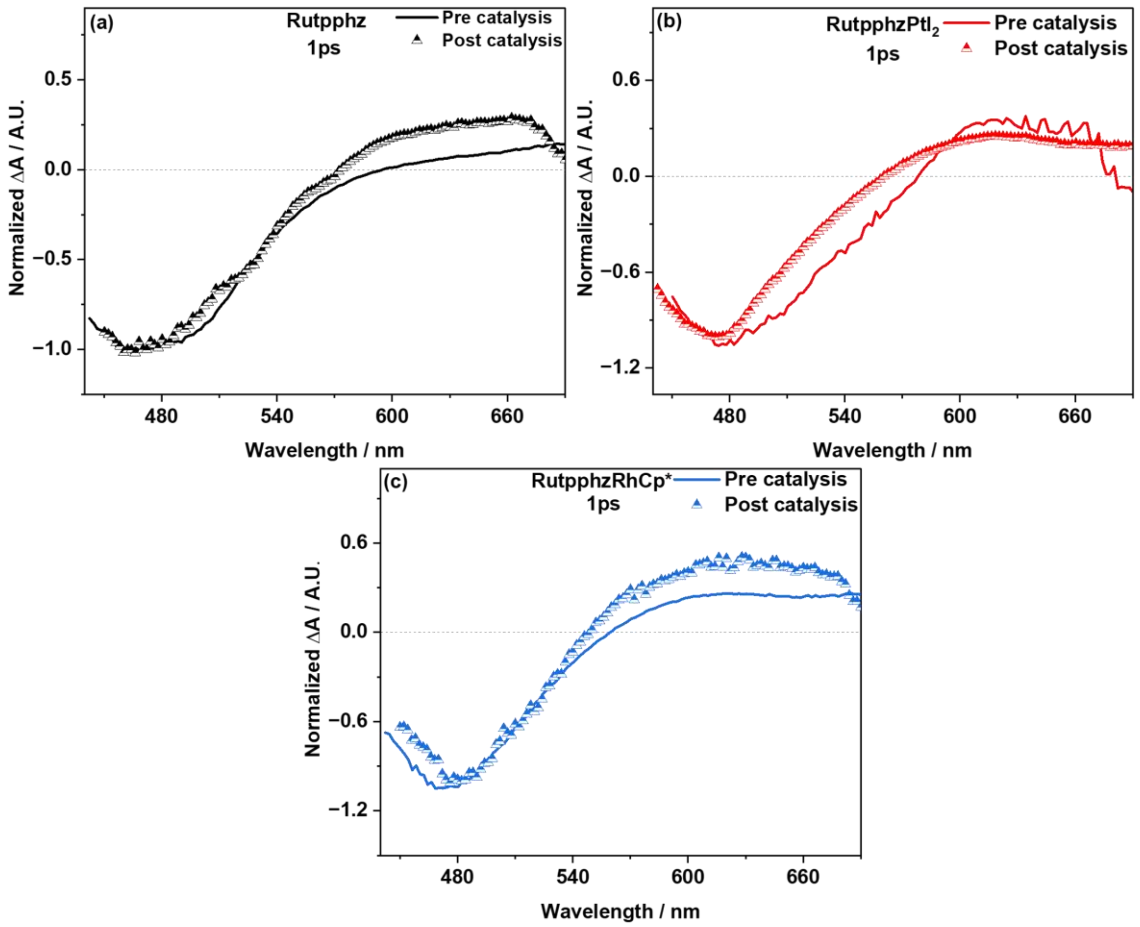

Figure S15 Normalized transient spectra of fibers with embedded Ru-complexes, before (solid line) and after (scatter plot) catalysis. The plotted spectra are at 1 ps delay time following excitation at 400 nm.

Hydrogen evolution reactions

Gas chromatography (GC) measurements for hydrogen quantification were performed on a Shimadzu GC-2030 equipped with a barrier ionization discharge detector (BID-2030), using helium as carrier gas. Separation was achieved with Restek SH-Rt-Molsieve 5 Å column (ID: 0.32 mm; film thickness: 30 µm, length: 30 m) operated at an oven temperature 80 °C. The GC was calibrated by direct injection of known amounts of standard hydrogen gas. If not stated otherwise 100 µL of the headspace were injected manually.

Aqueous sodium ascorbate solution used for catalytic experiments was degassed by flushing the solution with Ar for 2 h. For the reaction in vials 2 to 3 mg of fiber were placed into 4 mL GC vials and transferred into the glove box, were they were submerged in 2 mL of aqueous sodium ascorbate (100 mM). Illumination of the vials took place outside of the glove box using a reactor manufactured as described in [11]. Samples were illuminated at 450 nm with P = 0.08 W/cm², under rigorous ventilation to avoid build-up of heat. After 24 h the headspace of the solutions was analysed by GC. This procedure was repeated with the same fibers for three cycles in total. All experiments were performed as duplicates.

Table S9: Corrected TON of heterogeneous HER catalysis with the fibers in 100mM aqueous sodium ascorbate. Illumination at 450 nm for 24 h in boro-silicate vials.

Complex	TON (1. cycle, 24h)	TON (2. cycle, 48h)	TON (3. cycle, 72h)
Rutpphz	detected	detected	detected
$RutpphzPtI_2$	2.6	3.3	4.4
RutpphzRhCp*	1.2	1.4	1.7

For the reaction in the flow-reactor a 5 x 5 cm piece of the fibre was cut out and clamped into the irradiation window. Then the flow-reactor was assembled and flushed for 15 min with argon. 40 mL of aqueous sodium ascorbate (100 mM) was flushed with argon for 2 h to remove oxygen. The flow reactor was then filled with aqueous sodium ascorbate and the pump was started with a flow rate of 20 mL/h. After 24 h of illumination the amount of hydrogen was determined with GC.

Table S10: Corrected TON of heterogeneous HER catalysis with the fibers in 100mM aqueous sodium ascorbate. Illumination at 450 nm for 24 h in membrane flow-reactor.

Complex	TON
Rutpphz	detected
RutpphzPtI$_2$	7
RutpphzRhCp*	0.7

Homogeneous catalysis was performed under the same conditions as the experiments in the glass vials (see above). Catalytic solutions were prepared by dissolving the respective Ru-complex in MeCN. 100 µL of the MeCN stock solutions were then added to 2 mL of aqueous sodium ascorbate (100 mM). The final concentration is given in Table S11.

Table S11: TON of homogeneous HER catalysis with the Ru-complexes in 100mM aqueous sodium ascorbate. Illumination at 450 nm for 24 h in boro-silicate vials.

Complex	Concentration Ru-complex / µmol / L	TON
Rutpphz	59.1	detected
RutpphzPtI$_2$	48.7	7
RutpphzRhCp*	51.1	3

Ru-complexes under homogeneous catalytic conditions

Catalytic conditions were applied to the Ru-complexes, while the absorption spectra were monitored. Solutions were degassed by flushing argon through the solution for 2 h. The samples were prepared inside a glove box and irradiated with a 470 nm LED stick with simultaneous cooling through a fan. UV-vis spectra were taken with an Avantes AvaSpec-ULS2048CL detector unit coupled with an AVA AvaLight-DH-S-BAL light source. Light source, cuvette holder and detector were connected with fibreoptic cables. Measurements were performed in 1 cm quartz cuvettes.

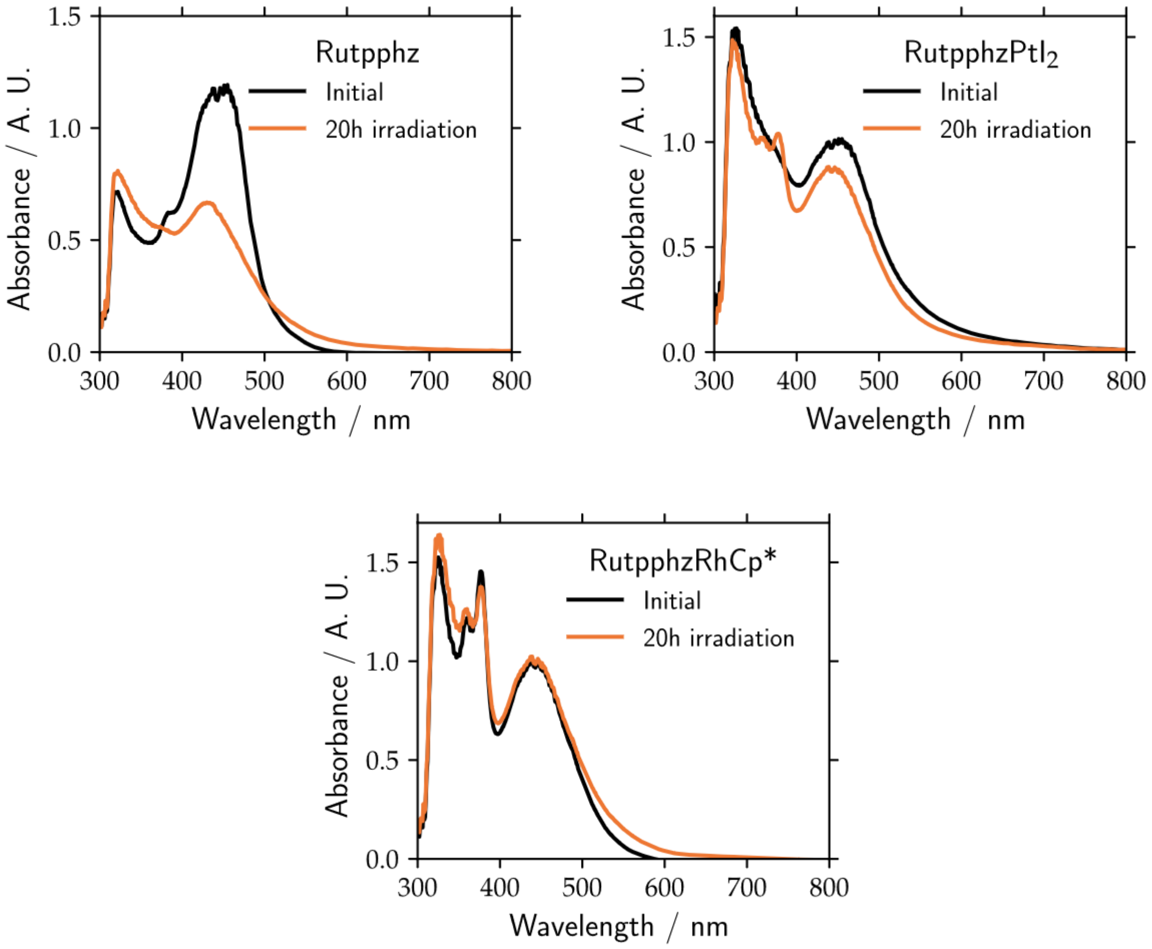

Figure S16: UV-vis spectra of the Ru-complexes before and after 20 h irradiation with 470 nm in aqueous sodium ascorbate.

Membrane reactor

The reactor setup consists of three main components: the reservoir, the pump, and the membrane reactor module, all connected by Cole-Parmer 1/8'' OD 1/16'' ID PVDF capillaries. The reservoir was equipped with two GL14 threads for BOLA HT laboratory screw joints to connect to the capillaries, as well as a Schlenk hose adapter to connect to an argon line. A KNF SIMDOS®10 FEM 1.10 TT.18 RC-P diaphragm liquid dosing pump was connected by Idex PEEK flangeless ¼-28 UNF flat bottom fittings.

The membrane reactor module contained the membrane reactor, a heatsink-mounted LED (ams OSRAM L4Z-40B208-0000 453 nm) and a 120 mm USB fan, all mounted onto a 3D printed holder.

The reactor was made from an aluminum framework and transparent FEP films from Bohlender, on which the electrospun fiber mat was placed, and a 5 mm PTFE spacer, assembled using M6x50 screws to create an enclosed volume of 50 mm x 50 mm x 5 mm = 12.5 mL. It was connected to the fluid circuit by the same Idex PEEK flangeless ¼-28 UNF flat bottom fittings as the pump.

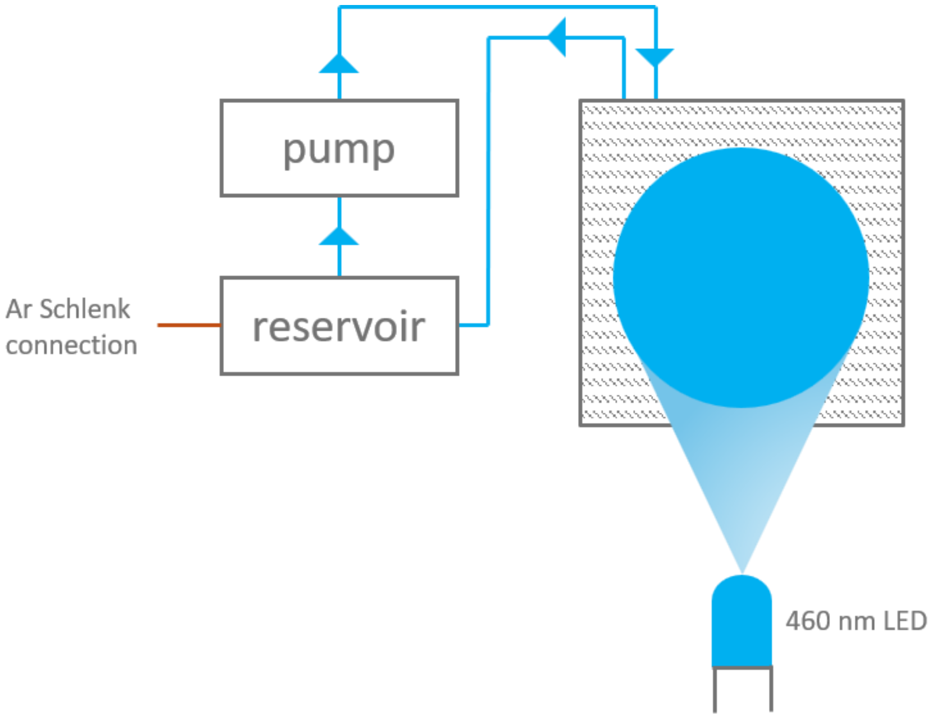

Figure S17: Schematic representation of the membrane reactor.

The LED was mounted on a black anodized S-series Ohmite heat sink (SA-LED-151E) with M3 screws and thermal paste and powered using a KORAD KA3005P benchtop power supply. The LED was characterized using an integrating sphere "Paula" from Mountain Photonics following the protocol reported by Sender *et al.*[12]

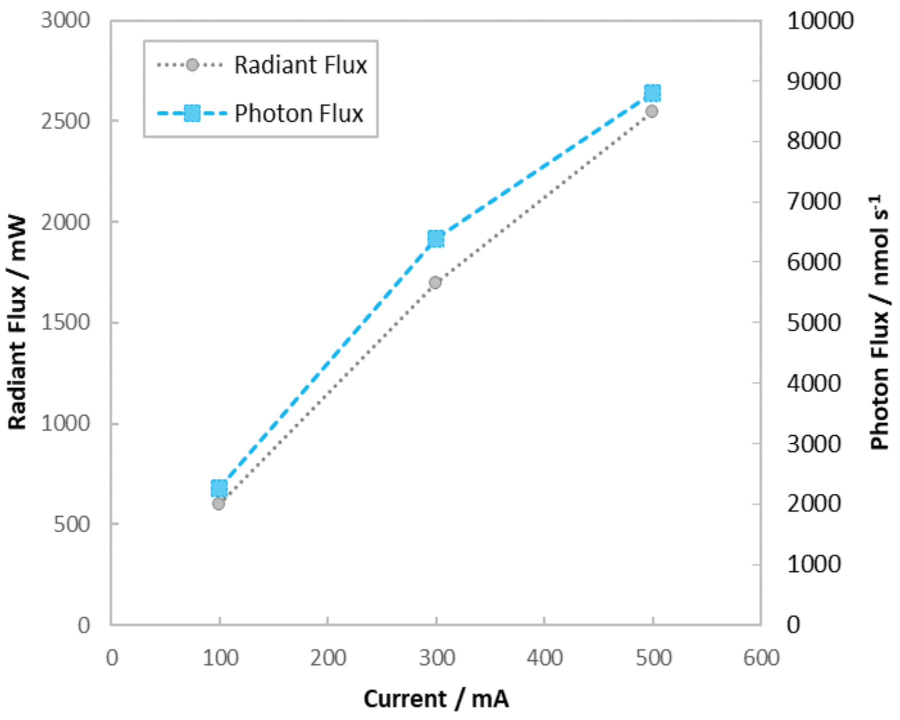

Figure S18: Radiometric measurement of the 453 nm LED used in the membrane reactor.

The heat sink was mounted onto the 3D printed module holder using M6x10 screws to keep the LED and membrane reactor in place and at a defined distance. The distance was chosen such that the circle projected onto the membrane reactor by the cone of light still achieves 50% of its peak intensity at the corners of the membrane to achieve overall high intensity. The module holder was printed using a Raise 3D Pro2 Plus with black Raise3D Premium PLA filament.

NMR spectra

^{31}P and ^{19}F spectra were taken to verify the integrity of the molecular components embedded into the fibers. Due to the higher sensitivity and higher concentration of fluorine atoms in the sample only the ^{19}F spectra are evaluated.

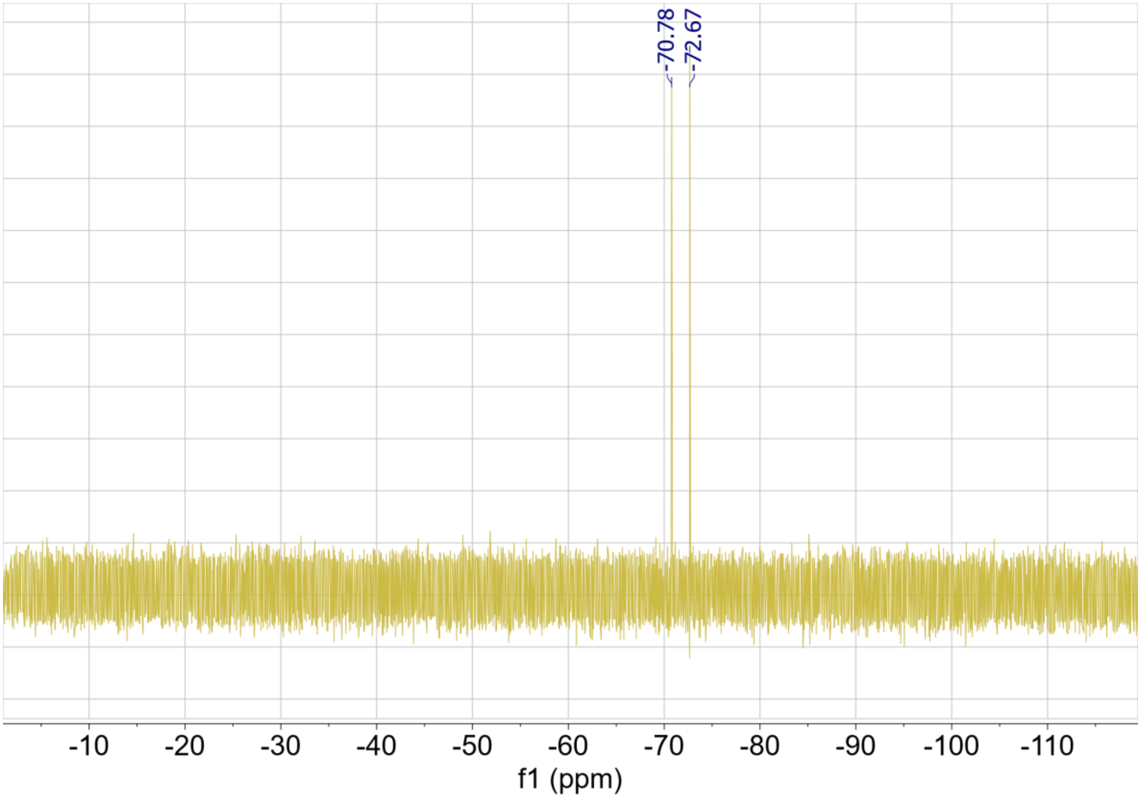

Figure S19: ^{19}F NMR spectrum (376 MHz) of Rutpphz dissolved in dmf-d^{7}.

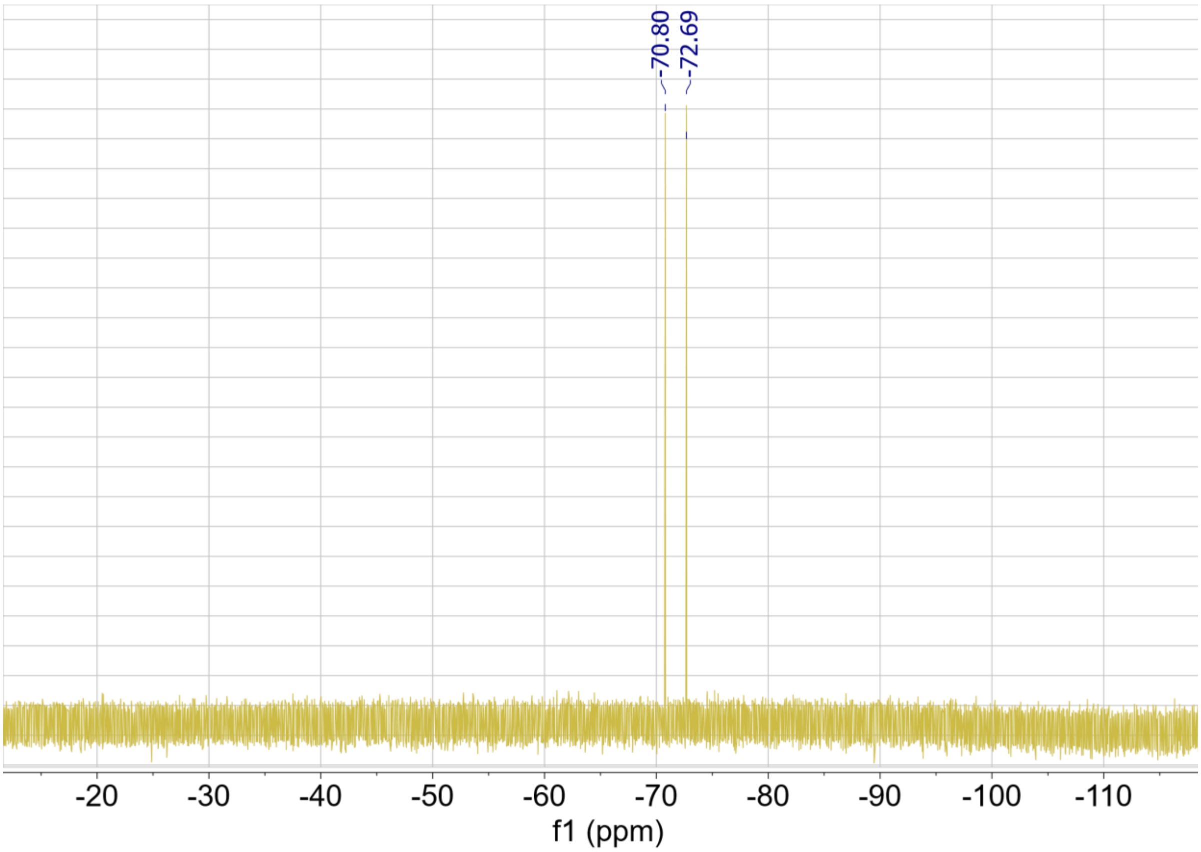

Figure S 20: 19F NMR spectrum (376 MHz) of RutpphzPtI2 dissolved in dmf-d7.

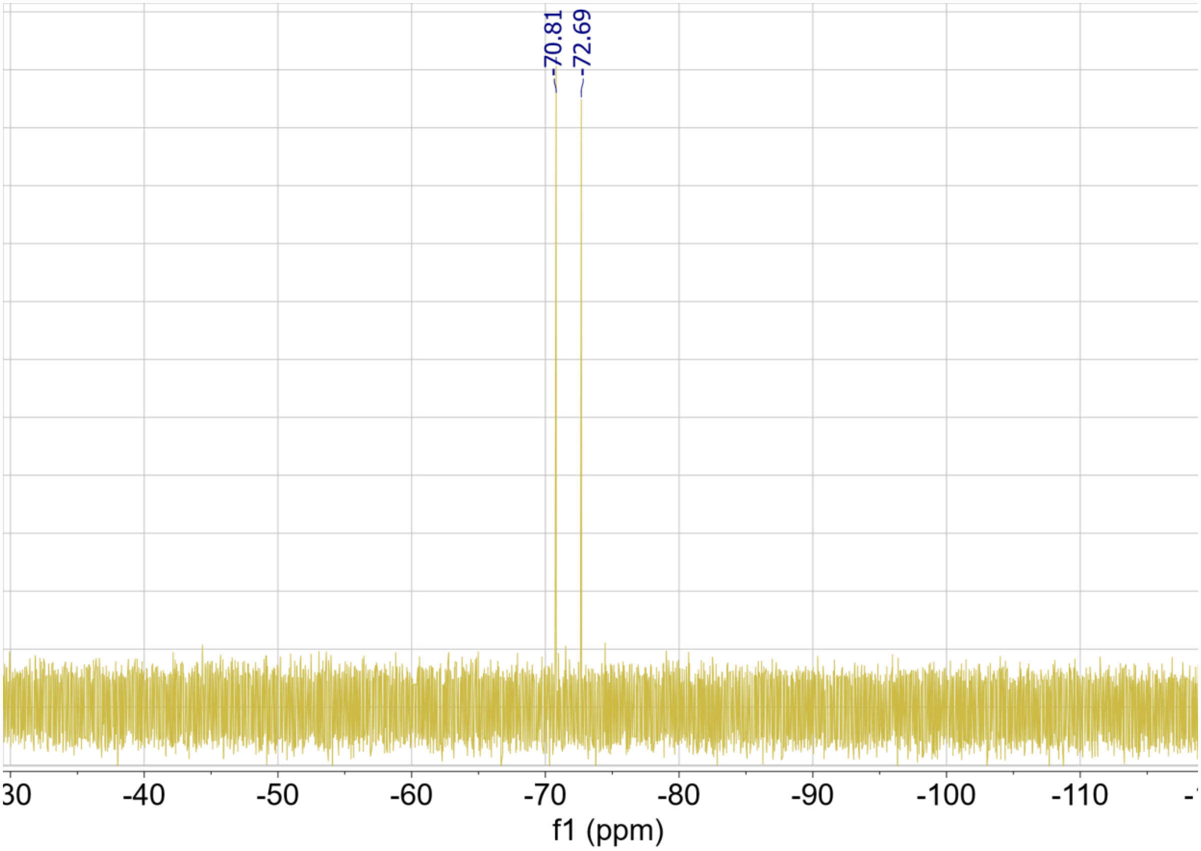

Figure S21: 19F NMR spectrum (376 MHz) of RutpphzRhCp* dissolved in dmf-d[7].

TA spectroscopy

Femtosecond transient absorption (fs-TA) spectra were captured using a pump-probe setup as previously described.[13] The setup consists of a Titanium-Sapphire (Ti:Sa) oscillator (Libra, Coherent Inc.),which generates ~ 100 fs pulses centered at 800 nm having a repetition rate of 1 kHz. A beam splitter divides this fundamental beam into two parts, one of which is utilized to generate the pump and the other to generate the probe. The excitation wavelength for the pump was obtained either via a beta-barium borate (BBO) crystal or an optical parametric amplifier (OPA). In this study, the sample was excited by a 400 nm pump beam generated from a BBO. We collected the data in UV-Vis region (350 nm – 700 nm) using a white light probe generated by a self-phase modulation of 800 nm beam in a rotating CaF_2 crystal. The pump and probe beams are spatially overlapped at the sample position, where their relative polarization is set to the magic angle of 54.7° by a Berek compensator and a polarizer. To observe time-dependent behavior, the arrival of the probe is delayed relative to the pump using a delay line. A charge-coupled device (CCD) array is used to measure the intensity change of the transmitted probe beam as a function of the delay time. Since the fiber samples are inherently opaque, we added a refractive index-matching solvent (microscopy grade immersion oil) to render them transparent and make measurements possible. Data collected is analyzed and fitted using the KiMoPack Python package.[14]

The Nanosecond transient absorption (ns-TA) and transient emission spectroscopy data were obtained using the previously described setup[15]. A Continuum OPO Plus pumped by a Continuum Surelite

Nd:YAG laser system (pulse duration 5 ns, repetition rate 10 Hz) generated the pump pulses at 450 nm. The probe light is provided by a 75 W Xenon arc lamp. The probe light is captured and analyzed with a Hamamatsu R928 photomultiplier, integrated with a detection system provided by Pascher Instruments AB.

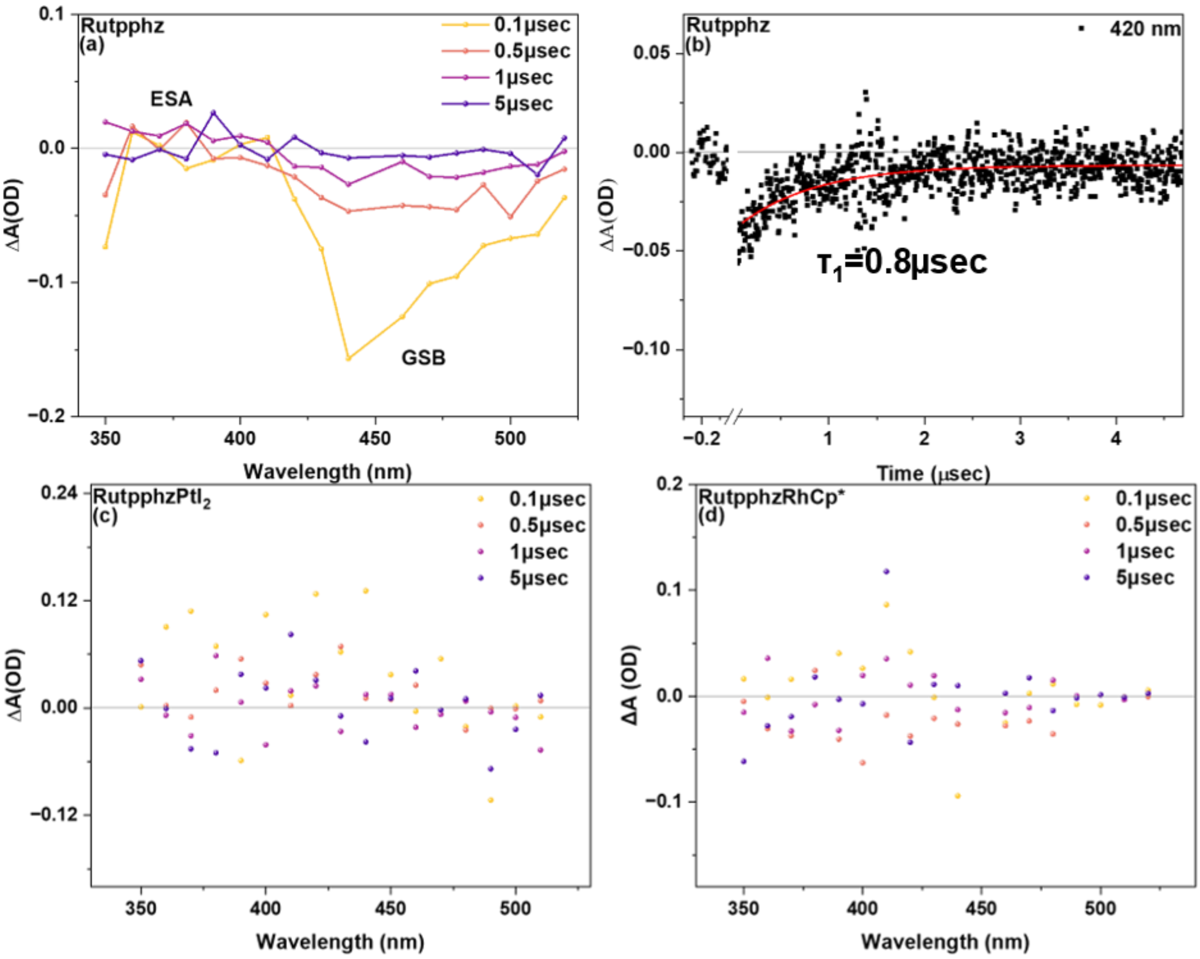

Figure S22: nsTA a) time dependent spectra and b) kinetics of Rutpphz and time dependent spectra of c) RutpphzPtI2 and d) RutpphzRhCp* in PAN fibers. The samples were excited with 450 nm pump.

References

(1) Mengele, A. K.; Kaufhold, S.; Streb, C.; Rau, S. Generation of a Stable Supramolecular Hydrogen Evolving Photocatalyst by Alteration of the Catalytic Center. *Dalton Trans.* **2016**, *45* (15), 6612–6618. https://doi.org/10.1039/C6DT00130K.

(2) Pfeffer, M. G.; Müller, C.; Kastl, E. T. E.; Mengele, A. K.; Bagemihl, B.; Fauth, S. S.; Habermehl, J.; Petermann, L.; Wächtler, M.; Schulz, M.; Chartrand, D.; Laverdière, F.; Seeber, P.; Kupfer, S.; Gräfe, S.; Hanan, G. S.; Vos, J. G.; Dietzek-Ivanšić, B.; Rau, S. Active Repair of a Dinuclear Photocatalyst for Visible-Light-Driven Hydrogen Production. *Nat. Chem.* **2022**, *14* (5), 500–506. https://doi.org/10.1038/s41557-021-00860-6.

(3) Bode-Aluko, C. A.; Pereao, O.; Ndayambaje, G.; Petrik, L. Adsorption of Toxic Metals on Modified Polyacrylonitrile Nanofibres: A Review. *Water. Air. Soil Pollut.* **2017**, *228* (1), 35. https://doi.org/10.1007/s11270-016-3222-3.
(4) Küllmer, M.; Herrmann-Westendorf, F.; Endres, P.; Götz, S.; Reza Rasouli, H.; Najafidehaghani, E.; Neumann, C.; Gläßner, R.; Kaiser, D.; Weimann, T.; Winter, A.; Schubert, U. S.; Dietzek-Ivanšić, B.; Turchanin, A. Two-Dimensional Photosensitizer Nanosheets via Low-Energy Electron Beam Induced Cross-Linking of Self-Assembled Ru^{II} Polypyridine Monolayers. *Angew. Chem.* **2022**, *134* (28), e202204953. https://doi.org/10.1002/ange.202204953.
(5) Lei, P.; Hedlund, M.; Lomoth, R.; Rensmo, H.; Johansson, O.; Hammarström, L. The Role of Colloid Formation in the Photoinduced H_2 Production with a $Ru^{II}-Pd^{II}$ Supramolecular Complex: A Study by GC, XPS, and TEM. *J. Am. Chem. Soc.* **2008**, *130* (1), 26–27. https://doi.org/10.1021/ja0776780.
(6) Horzum, N.; Shahwan, T.; Parlak, O.; Demir, M. M. Synthesis of Amidoximated Polyacrylonitrile Fibers and Its Application for Sorption of Aqueous Uranyl Ions under Continuous Flow. *Chem. Eng. J.* **2012**, *213*, 41–49. https://doi.org/10.1016/j.cej.2012.09.114.
(7) Zedler, L.; Guthmuller, J.; Rabelo De Moraes, I.; Kupfer, S.; Krieck, S.; Schmitt, M.; Popp, J.; Rau, S.; Dietzek, B. Resonance-Raman Spectro-Electrochemistry of Intermediates in Molecular Artificial Photosynthesis of Bimetallic Complexes. *Chem. Commun.* **2014**, *50* (40), 5227. https://doi.org/10.1039/c3cc47487a.
(8) Zedler, L.; Mengele, A. K.; Ziems, K. M.; Zhang, Y.; Wächtler, M.; Gräfe, S.; Pascher, T.; Rau, S.; Kupfer, S.; Dietzek, B. Unraveling the Light-Activated Reaction Mechanism in a Catalytically Competent Key Intermediate of a Multifunctional Molecular Catalyst for Artificial Photosynthesis. *Angew. Chem. Int. Ed.* **2019**, *58* (37), 13140–13148. https://doi.org/10.1002/anie.201907247.
(9) Yang, H.; Zhuang, G. V.; Ross, P. N. Thermal Stability of LiPF6 Salt and Li-Ion Battery Electrolytes Containing LiPF6. *J. Power Sources* **2006**, *161* (1), 573–579. https://doi.org/10.1016/j.jpowsour.2006.03.058.
(10) Zhuravlev, O. E.; Nikol’skii, V. M.; Voronchikhina, L. I. Thermal Stability of Quaternary Ammonium Hexafluorophosphates and Halides. *Russ. J. Appl. Chem.* **2013**, *86* (6), 824–830. https://doi.org/10.1134/S1070427213060062.
(11) Kowalczyk, D.; Li, P.; Abbas, A.; Eichhorn, J.; Buday, P.; Heiland, M.; Pannwitz, A.; Schacher, F. H.; Weigand, W.; Streb, C.; Ziegenbalg, D. Making Photocatalysis Comparable Using a Modular and Characterized Open-Source Photoreactor**. *ChemPhotoChem* **2022**, *6* (7), e202200044. https://doi.org/10.1002/cptc.202200044.
(12) Sender, M.; Ziegenbalg, D. Radiometric Measurement Techniques for In-Depth Characterization of Photoreactors – Part 2: 3 Dimensional and Integral Radiometry. *React. Chem. Eng.* **2021**, *6* (9), 1614–1627. https://doi.org/10.1039/D0RE00457J.
(13) ChemPhysChem - 2009 - Siebert - Spectroscopic Investigation of the Ultrafast Photoinduced Dynamics in -Conjugated.Pdf.
(14) Müller, C.; Pascher, T.; Eriksson, A.; Chabera, P.; Uhlig, J. KiMoPack: A Python Package for Kinetic Modeling of the Chemical Mechanism. *J. Phys. Chem. A* **2022**, *126* (25), 4087–4099. https://doi.org/10.1021/acs.jpca.2c00907.
(15) Stephenson, M.; Reichardt, C.; Pinto, M.; Wächtler, M.; Sainuddin, T.; Shi, G.; Yin, H.; Monro, S.; Sampson, E.; Dietzek, B.; McFarland, S. A. Ru(II) Dyads Derived from 2-(1-Pyrenyl)-1 *H* -Imidazo[4,5- *f*][1,10]Phenanthroline: Versatile Photosensitizers for Photodynamic Applications. *J. Phys. Chem. A* **2014**, *118* (45), 10507–10521. https://doi.org/10.1021/jp504330s.
